# Microemulsions of Essential Oils: Characterization and Phase Behavior

**DOI:** 10.3390/ijms27188165

**Published:** 2026-09-14

**Authors:** Dilek Gazolu-Rusanova, Zlatina Mitrinova, Ivan Lesov, Slavka Tcholakova

**Affiliations:** 1 Department of Chemical and Pharmaceutical Engineering, Faculty of Chemistry and Pharmacy, Sofia University, 1164 Sofia, Bulgaria; dg@lcpe.uni-sofia.bg (D.G.-R.); zm@lcpe.uni-sofia.bg (Z.M.); lesov@lcpe.uni-sofia.bg (I.L.); 2 Centre of Competence “Sustainable Utilization of Bio-Resources and Waste of Medicinal and Aromatic Plants for Innovative Bioactive Products” (BIORESOURCES BG), 1164 Sofia, Bulgaria

**Keywords:** microemulsions, solubilization, essential oils, preservatives

## Abstract

Essential oils are a key ingredient in foods, beverages, and personal- and home-care products due to their flavors, scents, and various biological activities. This study investigates the capacity of polysorbate 60 (PS60) micelles to solubilize eight essential oils classified into two groups based on their composition. The first group (tea tree, rose, lavender, and melissa oils) contains more than 40% polar aliphatic or alicyclic alcohols or aldehydes, whereas the oils from the second group (chamomile, yarrow, lemon, and orange oils) contain less than 20% of these compounds. The efficient solubilization of the studied essential oils within PS60 micelles is proposed to occur via a mechanism involving two steps: (1) the formation of mixed micelles between PS60 molecules and the polar alcohol or aldehyde constituents of the essential oils, and (2) the dehydration of PS60 ethoxy groups in the presence of highly water-soluble additives (such as citric acid and sodium citrate), which is suggested to decrease the hydrophilic barrier of the micellar corona, thereby facilitating the mass transfer of hydrophobic oil molecules into the micellar core. Stable, transparent microemulsions form only with the first group of essential oils in a certain concentration range. The second step in the proposed mechanism leads to the unexpected finding that reducing the concentration of water-soluble additives, such as citric acid and sodium citrate, impairs solubilization by allowing the micellar corona to rehydrate.

## 1. Introduction

Essential oils (EOs) are natural aroma compounds containing complex mixtures of primarily lipophilic components with various biological activities [1,2,3,4]. These oils are widely used in the pharmaceutical industry, perfumery, cosmetics, foods, and beverages. Among other properties, EOs are claimed to provide insecticidal, antioxidant, anti-inflammatory, antiallergic, antitumor, antibacterial, and antifungal activities [1,4,5,6,7,8,9,10,11,12,13,14,15,16].

To improve EO efficiency and perception, EOs are often delivered in the final products as micro- or nanoemulsions to allow [17]: (1) high bioavailability and chemical reactivity due to the higher oil–water interfacial area; (2) visual appeal through optical transparency; and (3) high stability against gravitational separation due to weak gravitational forces and Brownian motion effects.

Microemulsions are thermodynamically stable isotropic solutions that consist of water, oil, and a surfactant [18,19]. They are also called swollen micelles and form spontaneously under a narrow set of environmental conditions and compositions [19,20,21]. If these conditions are altered, then the microemulsion may no longer be thermodynamically stable and will be converted to another type of system, e.g., phase-separated, liquid crystalline, nanoemulsion, or conventional emulsion [22]. Macroemulsions are thermodynamically unstable systems in which one phase is dispersed in another phase as drops [19,21]. Microemulsions are usually easier to prepare than nanoemulsions and conventional emulsions because all phases (oil, water, and surfactant) are mixed homogeneously at a constant temperature [19,23]. The final equilibrium state of a thermodynamically stable microemulsion should, in principle, be independent of the preparation pathway [24], but the rate of microemulsion formation and the practical accessibility of the equilibrium can depend on the mixing protocol [25]. Much higher surfactant concentrations are required to prepare microemulsions in comparison to nanoemulsions because a comparable number of surfactant molecules is needed to solubilize the oil molecules in the swollen micelles [21]. Additives can affect the solubilization capacity of surfactant micelles based on their localization in the micelles or based on the dehydration of micellar associates and competition for water [26,27,28,29]. The most common co-surfactants used for micro- and nanoemulsion preparation are short/medium-chain-length alcohols, such as ethanol, glycerol, propylene glycol, polyethylene glycol derivatives, and sorbitol. They diffuse rapidly between the oil and water phases, reaching the interface. Some of these co-surfactants can intercalate between surfactant molecules, affecting both the polar heads and hydrocarbon tail interactions and changing the surfactant curvature. Due to their partitioning between the two phases, alcohols could also act as cosolvents and influence the solubility properties of the oil and water phases [30].

In a series of publications, Rao and McClements [20,31,32] show that the use of propylene glycol as an additive to lemon oil emulsions with polysorbates, sucrose palmitate, and others yields transparent flavor nanoemulsions with high stability. Propylene glycol is also used by Wulansari et al. [5] to facilitate the formation of tea tree oil nanoemulsions for pharmaceutical applications and by Souza et al. [33] for the preparation of orange oil microemulsions. In our previous study [34], we found that citric acid combined with sodium citrate, sodium benzoate, or potassium sorbate could be used for the preparation of transparent nanoemulsions of lemon oil and of a mixture of lemon and orange oil in the presence of polysorbate 60 or polysorbate 80 without using synthetic cosolvents such as propylene glycol. More recently, we discovered that the studied additives affect the micellar behavior of polysorbate 60 [35]. It was shown that polysorbate 60 forms elongated micelles upon the addition of a mixture of citric acid and sodium citrate. In these elongated micelles, the probability of interactions between the diester molecules (present as impurities) increases. As a consequence, upon storage, these molecules form lamellar phases, which precipitate and separate from the solution [35]. On the other hand, the intercalation of sodium benzoate or potassium sorbate into the palisade layer of polysorbate 60 micelles increases the area per molecule and decreases the packing parameter, leading to less elongated micelles. In these micelles, the diester molecules are evenly distributed and cannot be separated from the monoesters as lamellar phases. Therefore, potassium sorbate and sodium benzoate increase the solubilization of the diesters from polysorbate 60 and significantly improve the stability of the solutions against precipitation [35].

An in-depth literature search revealed a scarcity of information on additives, such as preservatives, that could facilitate microemulsion formation. This research extends the scope of our previous work [35], focusing on the effect of typical preservatives, such as citric acid (CA), potassium sorbate (KSorb), sodium benzoate (NaBenz), and a mixture of citric acid and sodium citrate (CA+NaCitr), on the formation and stability of microemulsions of lemon (LemO), orange (OrO), rose (RO), lavender (LavO), chamomile (CO), melissa (MO), tea tree (TTO), and yarrow (YO) essential oils (EOs) in a solution of polysorbate 60 (PS60). The major aims of the current study are to: (1) evaluate how the chemical composition of essential oils affects the solubilization capacity of PS60 micelles; (2) determine the effect of food additives on the solubilization of essential oils within PS60 micelles; and (3) elucidate the mechanism by which essential oil composition and aqueous phase composition govern the resulting dispersion type (microemulsions vs. macroemulsions). To achieve these aims, eight essential oils featuring distinct fractions of alcohols, aldehydes, esters, and hydrocarbons were investigated. Based on their composition, the oils were categorized into two groups: high-polar oils containing alcohols and aldehydes at ≥40 mol %, and low-polar oils containing alcohols and aldehydes at <20 mol % (Table S1). The effect of the aqueous phase was evaluated using various food additives (citric acid, potassium sorbate, sodium benzoate, and a mixture of citric acid and sodium citrate) in two experimental series: (1) at concentrations previously shown to form microemulsions with lemon oil upon heating to 90 °C [34], here evaluated at room temperature to assess ambient microemulsion formation; and (2) at a constant additive concentration in the aqueous phase to remove the effect of different water content at different additive concentrations. A combination of dynamic light scattering (DLS), small-angle X-ray scattering (SAXS), optical microscopy, and interfacial tension measurements was used to clarify the underlying mechanisms for the formation of microemulsions.

## 2. Results

### 2.1. Dispersions with 1 wt % EO

**Figure 1:**
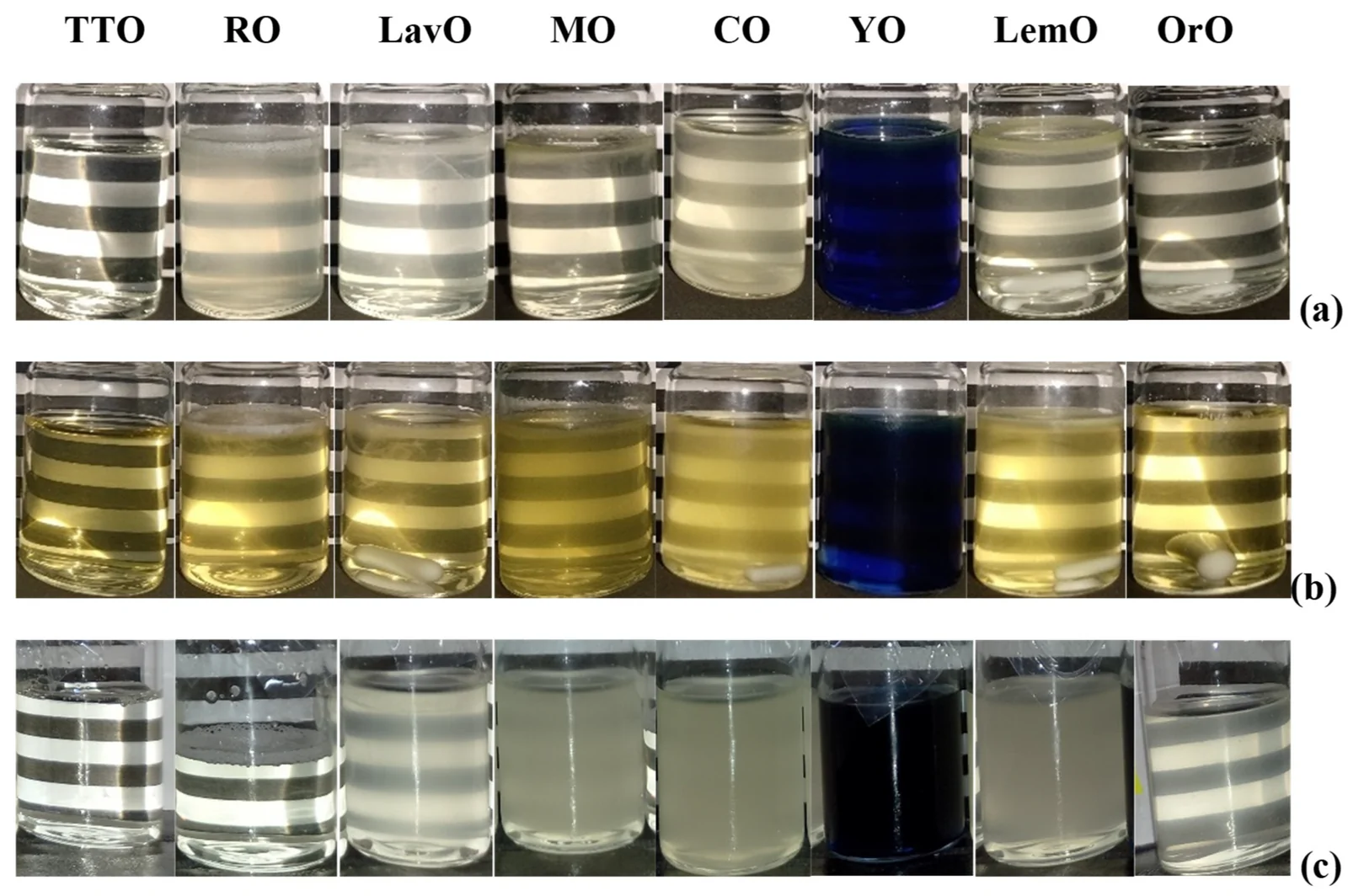
Visual appearance of mixtures of 1 wt % EO + 99 wt % aqueous phase, containing (**a**) 15 wt % PS 60, (**b**) 15 wt % PS 60 + 10 wt % KSorb, and (**c**) 15 wt % PS60 + 40 wt % CA + 4.7 wt % NaCitr, 1 day after their preparation.

Following the procedure described in Section 4.2, mixtures were prepared containing 1 wt % EO and 99 wt % of three different aqueous phases: PS60 (15 wt %), PS60+KSorb (15 wt % PS60 + 10 wt % KSorb), and PS60+CA+NaCitr (15 wt % PS60+40 wt % CA+4.7 wt % NaCitr). The illustrative pictures of the resulting dispersions, 1 day after their preparation, are shown in Figure 1.

**Figure 2:**
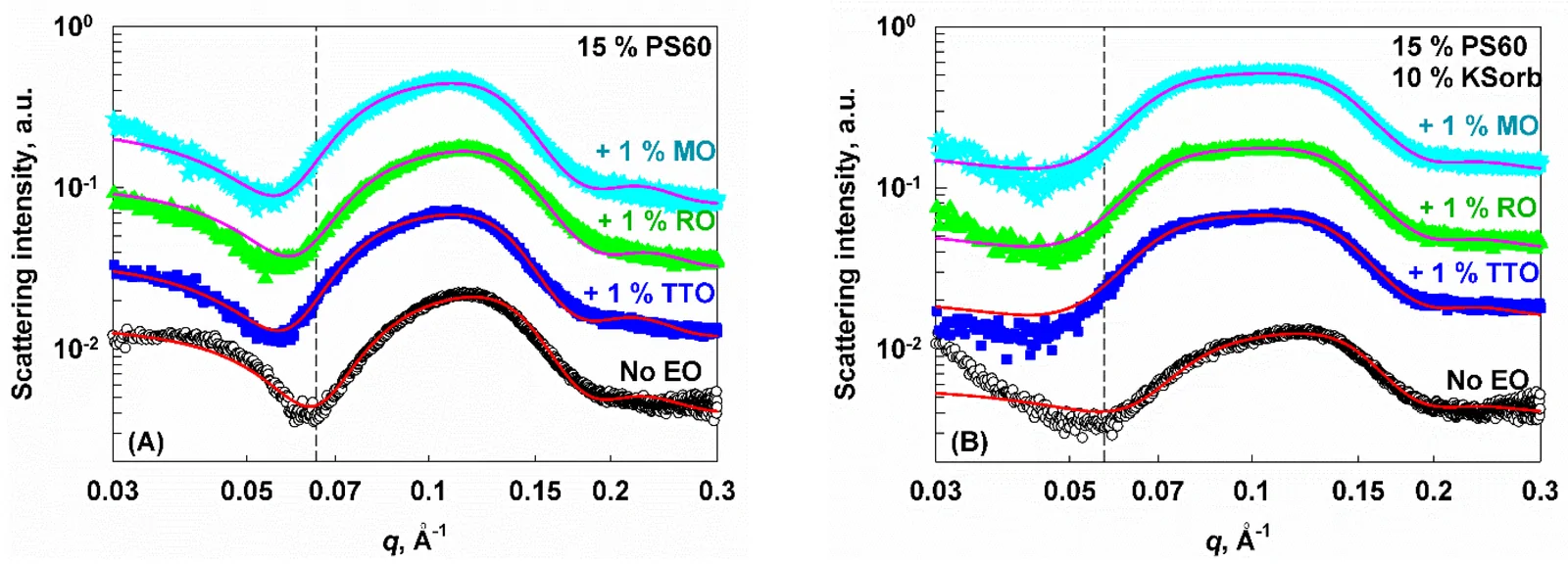
Scattering intensity as a function of the scattering vector, as measured by SAXS for dispersions containing 1 wt % EO in (**A**) 15 wt % PS60 and (**B**) 15 wt % PS60+10 wt % KSorb for TTO (blue squares), RO (green triangles), MO (cyan stars), and without added EO (black circles). For better illustration, the data are vertically shifted by a factor of 3. The red curves represent the best fit of the experimental data by using a core-shell ellipsoid model with the parameters shown in Table S3.

Tea tree oil (TTO) formed transparent dispersions with all three studied PS60 aqueous solutions, regardless of the presence of additives. The mixing procedure used leads to the formation of micrometer-sized drops, which are clearly visible under the microscope if the macroemulsion is formed. The fact that we do not see any objects under the microscope with TTO, and that dispersions remain stable and transparent over time (longer than a month), means that this oil forms microemulsions with all tested PS60 solutions. However, the rate of microemulsion formation significantly depends on the composition of the aqueous phase. Nearly instantaneous solubilization of TTO occurred in the PS60+CA+NaCitr solution (Table S2). In contrast, the time required to form a transparent microemulsion increased to 5 min when the PS60+KSorb solution was used, and to 15 min with the PS60 solution without additives. Faster microemulsion formation occurred in the system with the lowest water content (the PS60+CA+NaCitr solution contains only 39.3 wt % water, compared to 84 wt % water in the PS60 solution), showing that the TTO molecules partition between the aqueous and pseudo-micellar phases, which means that their incorporation into the micellar phase determines the rate of microemulsion formation. Following standard physicochemical terminology [19,36], we use the term oil solubilization to describe the incorporation of oily molecules into PS60 micelles. Below the saturation concentration, oily molecules localize within the micellar core or intercalate between the surfactant tails (Ref. [19], Figure 2), resulting in the spontaneous formation of a microemulsion. Once the oil concentration exceeds this saturation threshold, excess oil separates into micrometric drops, yielding the formation of the macroemulsion.

Rose oil (RO) formed macroemulsions when mixed with the PS60 or PS60+KSorb solutions. It formed a transparent dispersion only when mixed with the PS60+CA+NaCitr system, as shown in the second column of Figure 1. Achieving a clear dispersion with 1 wt % RO in this specific solution required approximately 20 h, which is much longer than the instantaneous solubilization observed for TTO. This prolonged equilibration time is probably driven by the chemical composition of RO, which contains long-chain hydrocarbons characterized by a high LogP ≈ 9.7 (Table S1) and negligible water solubility [36]. The mass transfer rate of long-chain hydrocarbons present in RO from mechanically generated drops into surfactant micelles is very slow, whereas the mass transfer of the primary hydrophobic component of TTO (γ-terpinene) is much faster, because γ-terpinene is a smaller molecule with non-zero water solubility that facilitates rapid micellar incorporation. The formation of the gel phase is observed upon storage of this dispersion, without visible drops.

The other essential oils used, lavender oil (LavO), melissa oil (MO), chamomile oil (CO), yarrow oil (YO), lemon oil (LemO), and orange oil (OrO), formed macroemulsions with all three tested aqueous solutions (PS60, PS60+KSorb, and PS60+CA+NaCitr). The formed oil drops float under gravity and form a cream layer at the top of the dispersions. The oil drops generated in the PS60+CA+NaCitr solutions were smaller than those in the other two solutions. Consequently, a fraction of these smaller drops remained dispersed throughout the bulk phase, causing persistent turbidity (Figure 1). Microscopic images of these emulsion drops are provided in Figure S1 of the Supporting Information. The drops from YO exhibited irregular, non-spherical shapes, which indicates the formation of an adsorption layer with high interfacial elasticity [37]. Because such rigid, elastic interfaces are not expected to be formed from low-molecular-weight non-ionic surfactants such as PS60 alone, the irregular shape of YO drops suggests the formation of a mixed-adsorption layer composed of both PS60 molecules and hydrophobic components from the YO, such as β-pinene, chamazulene, and β-caryophyllene (see Table S1).

To determine the effect of EOs on the shape of PS60 micelles, SAXS measurements were performed on dispersions containing 1 wt % of three studied EOs (TTO, RO, and MO) formulated in two different aqueous phases (15 wt % PS60 and 15 wt % PS60 + 10 wt % KSorb). The resulting spectra are presented in Figure 2. The specific type of EO has no significant impact on the scattering curves, despite noticeable differences in the macroscopic appearance of the samples (Figure 1). This indicates that the observed turbidity stems from an insoluble oily fraction that forms micrometer-sized droplets, whereas the solubilized fraction prompts the elongation of the PS60 micelles. The presence of the EOs slightly shifts the first scattering minimum toward smaller scattering vectors, showing the formation of larger micelles due to oil solubilization. To analyze the structures without the bias of model assumptions, an indirect Fourier transformation of the experimental scattering spectra was performed. The resulting curves, shown in Figure S2, reveal a typical core-shell structure characterized by consecutive maxima and minima, which arise from the scattering length density (SLD) contrast between the micellar core and shell.

**Figure 3:**
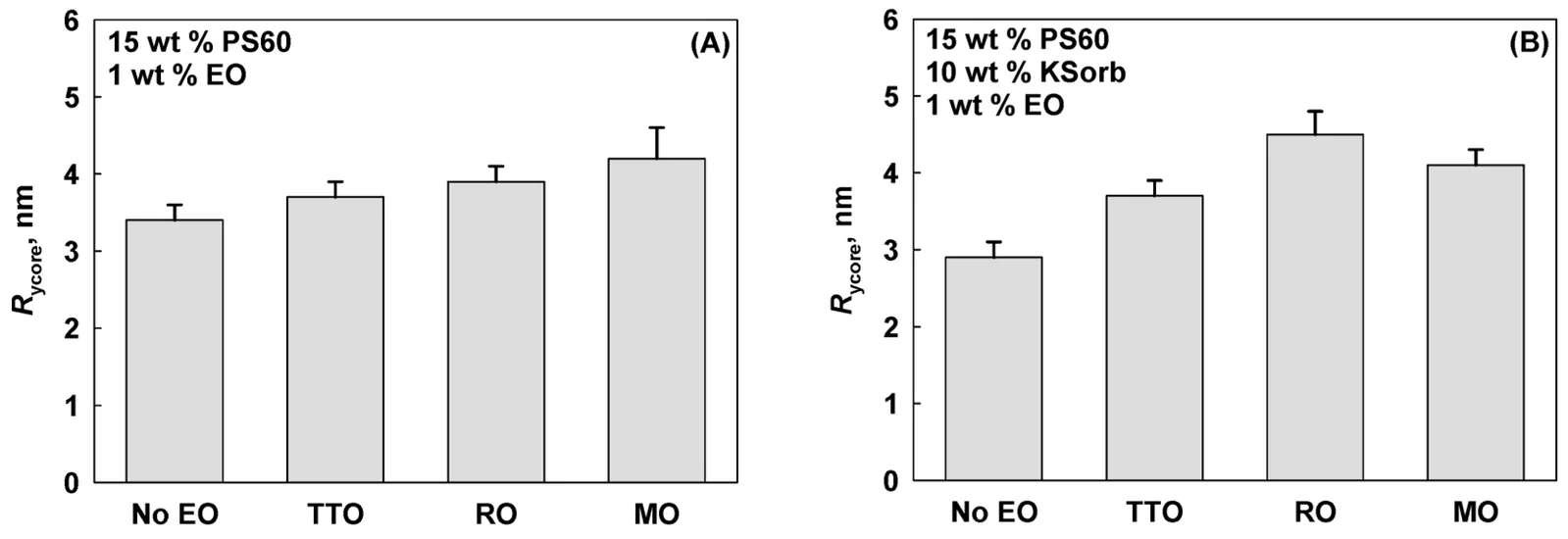
Polar core radius of the micelles formed in (**A**) 15 wt % PS60 and (**B**) 15 wt % PS60 +10 wt % KSorb solutions without and in the presence of 1 wt % EO, as determined from the best fit of SAXS spectra. The error bars denote model fitting uncertainties rather than experimental sample-to-sample variability.

To quantify the changes in PS60 micelles after EO addition, the experimental spectra were fitted using a previously shown core-shell ellipsoid model [35] to accurately describe PS60 micelles containing different preservatives. For the fitting procedure, the SLD values were fixed at 7.52 × 10^−6^ Å^−2^ for the hydrocarbon tails and at 10.4 × 10^−6^ Å^−2^ for the shell. The solvent SLD was set to 9.44 × 10^−6^ Å^−2^ for the 15 wt % PS60 solution and to 9.68 × 10^−6^ Å^−2^ for the 15 wt % PS60+10 wt % KSorb. A hard-sphere structure factor was incorporated to account for the excluded volume interactions of these non-ionic micelles. The ellipsoidal particle model utilizes two rotational axes: the equatorial core radius, *R*_xcore_, and the polar core radius, *R*_ycore_, assuming a uniform shell thickness across the entire ellipsoid [35]. The structural parameters obtained from the best fits are summarized in Table S3. The data demonstrate that EO addition does not significantly affect the equatorial core radius, *R*_xcore_. In contrast, the impact of the EOs on the polar core radius, *R*_ycore_, is very pronounced; see Figure 3. The incorporation of 1 wt % TTO into the 15 wt % PS60 solution increases *R*_ycore_ from 3.4 to 3.7 nm, whereas adding 1 wt % RO or MO leads to an increase to 3.9 and 4.2 nm, respectively. This elongation becomes even more pronounced for micelles formed in PS60+KSorb solutions, where *R*_ycore_ increases from 2.9 nm to 3.7 nm upon the addition of 1 wt % TTO, and up to 4.5 nm with 1 wt % RO and 1 wt % MO additions. The significant increase in the polar core radius upon the addition of TTO to PS60 and PS60+KSorb, determined from measured SAXS spectra, is in good agreement with the results obtained from DLS measurements, where a relatively small increase is observed in the size of micelles formed in the PS60 solution upon 1 wt % TTO addition, from 2.9 to 3.0 nm, whereas the effect of TTO is much more pronounced for micelles formed in PS60+KSorb solutions.

The primary mechanism driving this structural elongation is likely the intercalation of active oil components, alicyclic alcohols from TTO, aliphatic alcohols from RO, and aliphatic aldehydes and ketones from MO into the PS60 micelles. Because the headgroups of these alcohols, aldehydes, and ketones are significantly smaller than the bulky ethoxylated headgroups of PS60, their presence minimizes steric repulsion at the micellar surface, decreases the optimal area per headgroup, increases the packing parameter, and forces the micelles to elongate. Furthermore, the greater elongation observed with RO and MO compared to TTO is directly related to the structural geometry of their primary components. RO and MO primarily contain linear aliphatic alcohols and aldehydes, whose tails pack efficiently into PS60 micelles, whereas TTO contains alicyclic terpineol, which has high water solubility, and its partitioning within the micelles is lower.

From this series of experiments, we can conclude that the 15 wt % PS60 and 15 wt % PS60 + 10 wt % KSorb systems can effectively solubilize only the molecular components present in 1 wt % TTO. For all other studied essential oils, the solubilization capacity of these micelles is insufficient to maintain a single-phase system, resulting in the formation of macroemulsions rather than microemulsions. In contrast, the formulations containing 15 wt % PS60 + 40 wt % CA + 4.7 wt % NaCit successfully solubilize the components of both 1 wt % TTO and 1 wt % RO. However, the solubilization rate of the TTO components is significantly faster than that of the RO components. This kinetic difference is attributed to the lower water solubility of the n-paraffins found in RO compared to the higher water solubility of the alicyclic hydrocarbons present in TTO. The incorporation of aliphatic alcohols from RO, alongside aldehydes and ketones from MO, into the PS60 micelles significantly increases their polar core radius. This structural expansion suggests that stable microemulsions could potentially be achieved at even higher oil concentrations. This hypothesis was further explored using the 15 wt % PS60 + 40 wt % CA + 4.7 wt % NaCit system, which demonstrated the highest efficiency by completely solubilizing the components of both 1 wt % TTO and 1 wt % RO.

### 2.2. Solubilization Capacity of 15 wt % PS60 + 40 wt % CA + 4.7 wt % NaCitr

To estimate the solubilization limit of the different EOs, small portions of each oil were added in a stepwise manner to a solution containing 15 wt % PS60 + 40 wt % CA + 4.7 wt % NaCitr. The concentration ranges within which transparent microemulsions were obtained within 30 min after EO addition are summarized in Table 1. The mixing time of 30 min is estimated to be sufficient for the complete solubilization of a 20 µm oil drop into the aqueous phase under a diffusion-limited model [36], assuming an oil diffusion coefficient of 10^−10^ m^2^.s^−1^, a limiting aqueous solubility of 20 mM, and an oil molar volume of 170 mL/mol. Taking into account that mechanical stirring and the presence of PS60 micelles significantly accelerate mass transport, 30 min is expected to be more than adequate to reach thermodynamic equilibrium for most studied systems containing EOs with sufficiently high aqueous solubility. For EOs containing highly hydrophobic components with very low aqueous solubility, separate experiments were conducted with an extended equilibration time exceeding 24 h.

The PS60+CA+NaCitr system can accommodate up to 12.9 wt % TTO before macroemulsion formation occurs. Interestingly, adding RO at low weight fractions initially produces an opalescent dispersion. Note that in the previous section, the transparent microemulsion is formed with 1 wt % RO after 20 h. In the current experiment, the timescale is much shorter (30 min vs. 24 h), which is why the formulation containing 1 wt % RO was turbid, with micrometer-sized drops. However, further increases in RO concentration between 3.4 and 12.0 wt % lead to the formation of transparent microemulsions, indicating that all molecular components of RO become successfully solubilized within the micelles. This unusual behavior is most likely driven by the increased concentration of alcohols (such as ethanol, citronellol, geraniol, and nerol) at higher RO concentrations. These alcohols intercalate into the PS60 micelles, transforming them into elongated structures with a significantly higher capacity to accommodate n-paraffins. Because the fraction of aliphatic alcohols in RO is more than three times higher than that of n-paraffins (see Table S1), the components that enhance micellar solubilization capacity increase to a much greater extent than the hydrophobic molecules requiring solubilization. Macroemulsion formation is ultimately observed when the RO concentration exceeds 12 wt %, which is close to that of TTO.

A similar trend was observed for LavO and MO. While 1 wt % LavO remains as micrometer-sized emulsion drops when mixed with the PS60+CA+NaCitr solution, even after 24 h, it successfully forms a stable microemulsion when added within a concentration range between 4.9 wt % and 6.2 wt %. Similarly, MO unexpectedly forms microemulsions within the range of 5.5 wt % to 6.8 wt %. As shown by the structural data in Table S3 and Figure 3, even the addition of 1 wt % MO significantly expands the polar core radius of the PS60 micelles. However, this structural adjustment is insufficient to solubilize all of the oil’s hydrophobic components under those specific conditions. At 1 wt % LavO, the total molar fraction of linalool and linalyl acetate (the primary components of LavO) within the PS60 micelles is only 20%. This fraction increases to 40% when 5 wt % LavO is added, producing mixed micelles that are far more effective at co-solubilizing the remaining hydrophobic ingredients of the oil. Correspondingly, for MO, the aldehydes (geranial, neral, and citronellal) comprise only 10 mol % of the mixed micelles when 1 wt % MO is added, but increase to 30 mol % at a 5 wt % oil concentration. The aliphatic alcohols and aldehydes from RO, LavO, and MO form mixed aggregates with PS60 in the presence of CA+NaCitr that systematically boost solubilization capacity for their respective hydrophobic fractions, establishing a distinct concentration window where transparent microemulsions can exist.

In contrast, transparent microemulsions could not be achieved with CO, YO, LemO, or OrO across the entire tested concentration range. As indicated in Table S1, the molar fractions of alcohols and aldehydes in these specific EOs are very low. Their addition does not lead to the formation of mixed micelles capable of hosting the high fractions of alicyclic hydrocarbons that dominate these oils. For instance, although LemO contains aldehydes and ketones, their molar fraction within the mixed PS60 micelles remains below 15% even after 5 wt % oil addition, which is insufficient to facilitate the incorporation of the more hydrophobic molecules. Macroemulsions form across the entire concentration spectrum for these oils, and the turbidity of the resulting emulsions increases upon storage (Figure S3). This instability is attributed to the precipitation of PS60 diesters, as evidenced by the lamellar phases observed under polarized light (Figure S3C). While the components of CO, YO, LemO, and OrO do not prevent diester precipitation, the alcohols and aldehydes supplied by TTO, RO, LavO, and MO effectively suppress it, ensuring the long-term stability of formed microemulsions.

Experiments in which we re-evaluated the solubilization range after a 24 h equilibration period were also performed. The obtained experimental results shown in Table 1 reveal that there is no significant difference in the concentration range in which transparent microemulsions are formed with LavO and MO, determined after 30 min and 24 h equilibration periods. The only noticeable difference was observed for RO, which contains long-chain hydrocarbons. Due to their extremely low aqueous solubility, the required time to prepare a transparent microemulsion at 1 wt % RO is around 20 h. Note that an increase in the concentration of RO increases the amount of alcohols and aldehydes in the formulation and decreases the time required for the formation of a transparent microemulsion; as a consequence, there is no change in the highest concentration of RO that can be incorporated into the micelles between storage times of 30 min and 24 h.

### 2.3. Dispersions Formed with 5 wt % Essential Oil

**Figure 4:**
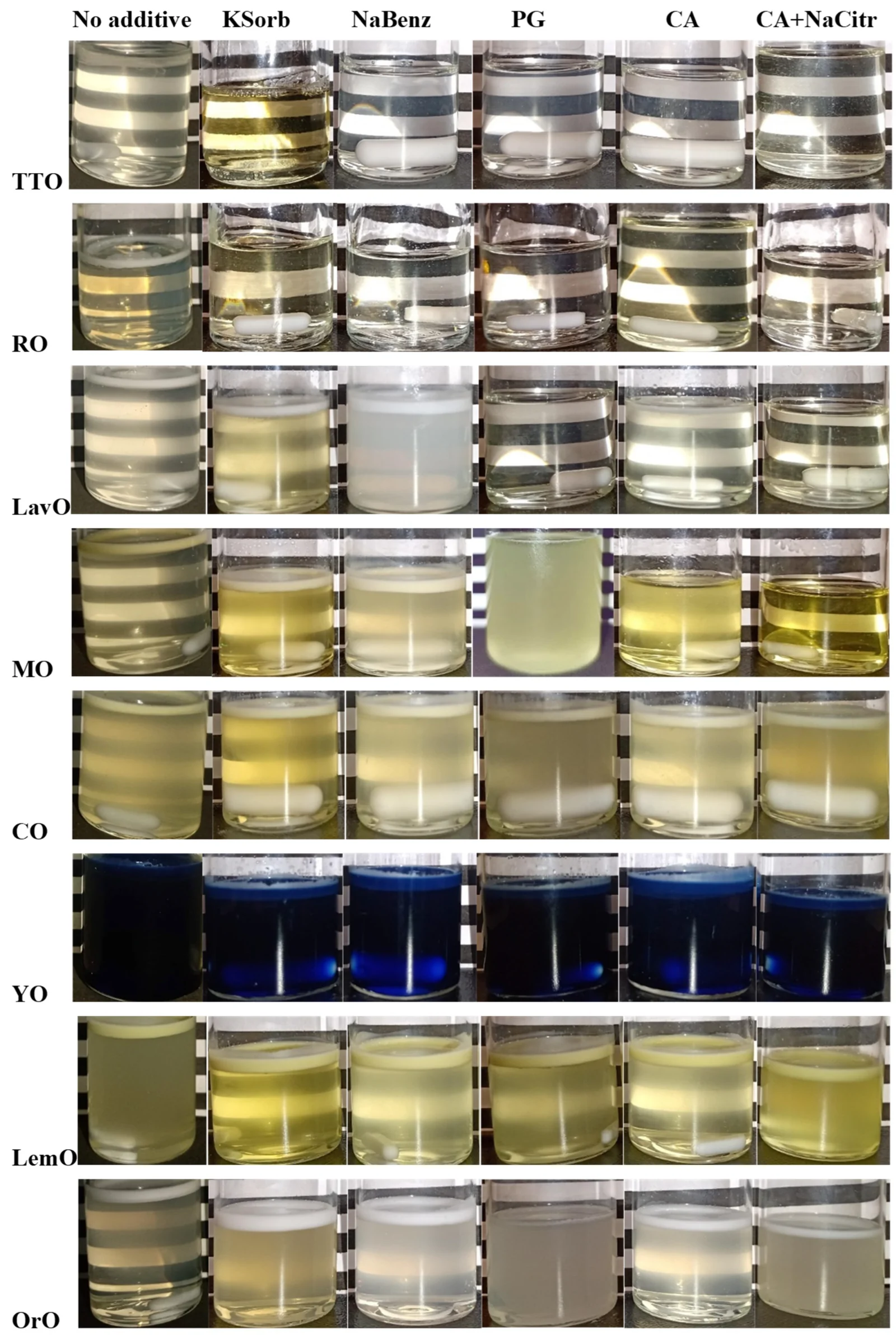
Picture taken 24 h after dispersion preparation with 5 wt % EO in 15 wt % PS60 (first column); 15 wt % PS60+10 wt % KSorb (second column); 15 wt % PS60+10 wt % NaBenz (third column); 15 wt % PS60+28.3 wt % PG (forth column); 15 wt % PS60+20 wt % CA (fifth column); and 15 wt % PS60+40 wt % CA+4.7 wt % NaCitr (sixth column).

To determine how different food additives affect the solubilization capacity of 15 wt % PS60 solutions, a series of experiments was conducted by introducing 5 wt % of various EOs. The concentration of 5 wt % was chosen because this is the concentration at which the microemulsions are formed, not only with TTO but also with RO, MO, and LavO, as can be seen in Table 1. These oils were added to the 15 wt % PS60 solution without additives, as well as to PS60 solutions with either 10 wt % KSorb, 10 wt % NaBenz, 20 wt % CA, 28.3 wt % PG, or a combination of 40 wt % CA and 4.7 wt % NaCitr. The resulting dispersions are shown in Figure 4.

The PS60 solution without additives proved incapable of completely solubilizing any of the studied EOs at a 5 wt % concentration. Even TTO, which can be accommodated up to 12.9 wt % in the 15 wt % PS60 + 40 wt % CA + 4.7 wt % NaCitr system, cannot be solubilized at 5 wt % in 15 wt % PS60 solution without additives, yielding a turbid macroemulsion instead. This demonstrates that while PS60 micelles can successfully incorporate 1 wt % TTO, they cannot accommodate 5 wt % TTO. Consequently, the increasing molar fraction of terpinen-4-ol (the main component of TTO) within the PS60 micelles is insufficient on its own to expand their capacity to host co-existing alicyclic hydrocarbons, such as γ-terpinene.

In contrast, all 15 wt % PS60 solutions containing any of the tested additives successfully solubilized 5 wt % TTO, resulting in transparent, stable microemulsions (Figure 4). However, the time required to achieve full transparency varied significantly depending on the additive used (see Table S4). Equilibration required only 2 min when 28.3 wt % PG or 40 wt % CA + 4.7 wt % NaCitr were added, but extended to 15 min with 10 wt % KSorb and to 55 min with 20 wt % CA. The slowest solubilization occurred with 10 wt % NaBenz, taking up to 110 min. This pronounced kinetic variation indicates that the initial size and structure of the PS60 micelles formed in the presence of different additives significantly affect the rate of TTO solubilization. In our previous study [35], we demonstrated that elongated micelles are formed in the presence of 40 wt % CA + 4.7 wt % NaCitr, which explains why the rate of TTO incorporation into these structural assemblies is much faster compared to systems with other additives.

The behavior of the dispersions containing 5 wt % RO is very similar to that of the TTO systems. While the pure 15 wt % PS60 solution could not solubilize 5 wt % RO, the presence of any studied additive successfully boosted the micellar solubilization capacity, leading to transparent microemulsions. Interestingly, the kinetics of microemulsion formation were faster for RO than for TTO across all tested additives. Instantaneous solubilization was achieved using 28.3 wt % PG or 40 wt % CA + 4.7 wt % NaCitr. The equilibration time increased to 11 min for both 10 wt % KSorb and 20 wt % CA, and reached 55 min when 10 wt % NaBenz was added.

For systems containing 5 wt % LavO, formulation requirements became considerably more restrictive. Clear microemulsions were successfully achieved only in the systems containing 28.3 wt % PG or 40 wt % CA + 4.7 wt % NaCitr. Furthermore, the solubilization kinetics for LavO slowed down substantially, requiring 15 min to reach clarity compared to the instantaneous or 2 min equilibration observed for RO and TTO, respectively. All other evaluated additives do not form micelles that can incorporate the full spectrum of LavO components, resulting in macroemulsions.

The formulation constraints were narrowest for 5 wt % MO, where transparent microemulsions were exclusively obtained using the 15 wt % PS60 + 40 wt % CA + 4.7 wt % NaCitr system, requiring an equilibration period of 13 min. All other tested additives, including PG, were ineffective at solubilizing MO at this concentration.

In agreement with the trends observed at the 1 wt % EO concentration, increasing the oil concentration to 5 wt % for the remaining EOs (CO, YO, LemO, and OrO) led to the formation of macroemulsions. This behavior is attributed to the lower percentage of amphiphilic alcohols and aldehydes in these specific EOs. As shown in the photographs in Figure 4, these oils form macroemulsions characterized by relatively large drops. Due to these large drops and density mismatch, these systems undergo rapid creaming. It is also worth noting that upon storage, a portion of the paraffins present in RO separated into a gel-like phase at the top of the macroemulsions after several days at room temperature.

The pH of the 15 wt % PS60 + 40 wt % CA + 4.7 wt % NaCitr solution was measured to be 2.2. To determine whether this low pH was the primary driving factor for the enhanced solubilization capacity of the 15 wt % PS60 + 40 wt % CA + 4.7 wt % NaCitr solution, we mixed 5 wt % TTO with a 15 wt % PS60 solution, which was adjusted to pH 2 using a 6 M HCl solution. The resulting emulsion exhibited a similar opalescent visual appearance to the 5 wt % TTO emulsion in the 15 wt % PS60 solution at its natural pH of 6.8. This control result confirms that the improved solubilization of hydrophobic compounds from TTO in PS60 micelles in the presence of 40 wt % CA + 4.7 wt % NaCitr is driven directly by the modifications of PS60 micelles induced by the citric acid and sodium citrate mixture, rather than a simple pH effect, enabling the successful preparation of stable microemulsions with EOs containing a sufficiently high molar fraction of alcohols and aldehydes.

Based on this series of experiments, it can be concluded that the 15 wt % PS60 + 40 wt % CA + 4.7 wt % NaCitr system is the most efficient for inducing microemulsion formation with the studied essential oils, followed closely by the 15 wt % PS60 + 28.3 wt % PG solution. An intermediate solubilization efficiency is exhibited by the 15 wt % PS60 + 10 wt % KSorb, 15 wt % PS60 + 10 wt % NaBenz, and 15 wt % PS60 + 20 wt % CA systems. In contrast, the 15 wt % PS60 solution without additives proved to be the least efficient, as it was entirely unable to solubilize any of the tested essential oils at a 5 wt % concentration.

To determine how the increased concentration of EOs in 15 wt % PS60 and 15 wt % PS60 + 10 wt % KSorb affects the resulting micellar aggregates, DLS and SAXS measurements were performed using two representative oils: TTO and RO. TTO forms a microemulsion at 1 wt % in both solutions, but at 5 wt %, it can be completely solubilized only in the presence of 10 wt % KSorb. On the other hand, RO forms a microemulsion at 5 wt % only in the 15 wt % PS60 + 10 wt % KSorb system, while yielding macroemulsions at 1 wt % in both solutions. SAXS measurements were also extended to all studied EOs mixed with the 15 wt % PS60 + 40 wt % CA + 4.7 wt % NaCitr solution (see Table S5).

Initially, the average hydrodynamic diameters of the micelles were measured using DLS. For the 15 wt % PS + 10 wt % KSorb solution, the mean volume diameter increased from 6 ± 0.3 nm (no EO) to 8.2 ± 0.2 nm upon the addition of 5 wt % TTO. The obtained SAXS spectra are presented in Figure S4, plotted along with the respective SAXS spectra of the reference solutions without EOs. The scattering intensities at 5 wt % EO differ significantly from those obtained at 1 wt % EO, indicating that the incorporation of EO components into the PS60 micelles fundamentally alters their shape and size. Modeling the experimental data using a prolate core-shell ellipsoid model under the assumption that the scattering length densities (SLDs) of the core, shell, and solvent remain constant upon oil incorporation shows an increase in the equatorial core radius from 1.9 nm to 2.9 nm, and a corresponding increase in the polar core radius from 2.9 nm to 5.6 nm upon the addition of 5 wt % TTO in the 15 wt % PS + 10 wt % KSorb solution (Table S5). This significant change in the SAXS profiles unambiguously demonstrates that the EO molecules partition between the aqueous phase and PS60 micelles, changing their structure.

A close look at the fitting error for the prolate core-shell ellipsoid model showed that it increased from 3.3 × 10^−7^ (no EO) to 2.2 × 10^−6^ (5 wt % TTO), primarily because the model failed to accurately capture the *q*-value region between 0.1 and 0.3 Å^−1^. To address this, the experimental data were refit using an oblate core-shell ellipsoidal geometry. The oblate model yielded a better description of the high-q values, reducing the net fitting error two-fold for microemulsions formed with 5 wt % EOs (see Table S5).

The parameters derived from the optimized oblate core-shell ellipsoid fits for systems containing 5 wt % EO are shown in Table 2, and a direct comparison between the prolate and oblate models is provided in Table S5. Both models converge on highly consistent values for the effective micellar radius, shell thickness, and volume fraction. In 15 wt % PS60 solutions, the effective micellar radius increases from 4.7 nm (at 1 wt % TTO) to 6.4 nm (at 5 wt % TTO). Although 5 wt % TTO exceeds the equilibrium solubilization capacity of the pure surfactant solution and triggers macroemulsion phase separation, a significant fraction of the oil still partitions into the micellar phase, driving this change in size and shape. Interestingly, the shell thickness remains practically unaltered under these conditions, implying that the alicyclic alcohols and hydrocarbons of TTO do not significantly perturb the hydrophilic corona formed by the polyoxyethylene headgroups of PS60.

In contrast, increasing the RO concentration from 1 wt % to 5 wt % in PS60 leads not only to a noticeable increase in the effective radius from 4.5 nm to 5.5 nm but also to a reduction in shell thickness from 1.6 nm to 1.4 nm. Similar trends are observed when using the 15 wt % PS60 + 10 wt % KSorb solution, where both oils form fully transparent microemulsions. Here, the effective radius for TTO increases from 4.4 nm (1 wt % TTO) to 5.5 nm (5 wt % TTO), with no significant alteration to the shell thickness, reinforcing that TTO components do not compromise the shell layer even in the presence of KSorb. On the other hand, raising the RO concentration to 5 wt % expands the effective radius from 4.4 nm to 5.3 nm while simultaneously compressing the shell thickness from 1.6 nm to 1.2 nm. This indicates that the aliphatic alcohols from RO alter the swelling behavior of the polyoxyethylene headgroups.

The incorporation of 5 wt % EO into the highly efficient 15 wt % PS60 + 40 wt % CA + 4.7 wt % NaCitr system universally results in oblate ellipsoidal micelles with effective radii ranging between 5.5 nm (TTO) and 6.7 nm (OrO). All essential oils that are fully solubilized within this solution and form a microemulsion have an effective radius below 6.1 nm, whereas the EOs that form macroemulsions exhibit swollen micelles in equilibrium with micro-sized drops, yielding an effective radius above 6.1 nm (Table 2). Furthermore, the micellar volume fractions determined from the best fits are somewhat lower for the systems in which the whole EOs are successfully solubilized in the micelles compared to the phase-separated macroemulsions. This difference stems from the fact that the poorly solubilized EOs contain a significantly lower proportion of amphiphilic alcohols and aldehydes capable of co-solubilizing the bulk alicyclic and aliphatic hydrocarbons present in the oil phase. As a consequence, these hydrophobic components are solubilized in the core of the micelles and increase their size, although parts of these components remain dispersed as oily drops in the solution.

It should be noted that the results presented in Table 2 were initially derived under the idealized assumption that the hydrophobic core consists solely of the aliphatic tails of the PS60 molecules, despite the fact that surfactant molecules make up only about 30% of the micellar mass at 5 wt % EO loading. To account for the modified electron density profile caused by EO incorporation in PS60 micelles, the SLD of the core was re-estimated for the 5 wt % TTO system based on LogP-driven phase partitioning (Table S1). Under the assumption that the partitioned oil molecules reside strictly within the hydrophobic core, the calculated core SLD shifts from 7.52 × 10^−6^ Å^−2^ to 8.09 × 10^−6^ Å^−2^. Refitting the experimental scattering data with this updated core SLD revealed no significant discrepancies in the primary geometric parameters. The only noticeable shift was a minor decrease in the calculated shell thickness, which changed from 2.0 nm to 1.5 nm for the 15 wt % PS60+5wt % TTO dispersion and from 1.6 nm to 1.3 nm for the 15 wt % PS60 + 10 wt % KSorb+5wt % TTO microemulsion (Table 2). Consequently, we can conclude that the compositional drift of the micellar core upon EO solubilization changes the bulk core SLD only to a minor extent, confirming that treating the core SLD as equivalent to pure PS60 provides sufficiently robust results.

## 3. Discussion

From the performed experiments, it can be seen that PS60 micelles without additives are unable to solubilize any of the studied 5 wt % EOs, including those containing a significant fraction of amphiphilic alcohols, such as TTO and RO. While SAXS measurements reveal significant alterations in micellar size and shape, the inherent solubilization capacity of the pure PS60 micelles is insufficient to host the entire hydrophobic components in these EOs, resulting in macroemulsion formation. The addition of 10 wt % KSorb to the 15 wt % PS60 solution enables the formation of clear microemulsions with the alcohol-rich EOs (TTO and RO). The primary difference between micelles formed with 5 wt % TTO or 5 wt % RO with and without 10 wt % KSorb lies in the smaller effective micellar radius and higher volume fraction observed in the presence of KSorb. This higher volume fraction is anticipated because a substantial portion of the TTO and RO molecules fails to incorporate into the PS60 micelles when KSorb is absent. The smaller effective radius in the presence of KSorb is likely related to a compressed shell thickness, which subsequently dampens intermicellar steric interactions.

Introducing 40 wt % CA and 4.7 wt % NaCitr to the 15 wt % PS60 system reduces the shell thickness even further (Table 2). Consequently, these highly concentrated solutions induce microemulsion formation not only with TTO and RO, but also with LavO and MO. As demonstrated in our previous study [35], this severe compression of the PS60 shell layer upon the addition of 40 wt % CA and 4.7 wt % NaCit stems from a significant reduction in the number of free water molecules available to hydrate the polyoxyethylene headgroups, resulting in a significantly dehydrated corona. This dehydrated shell environment lowers the thermodynamic barrier, facilitating the migration of EO components into the micellar core and microemulsion formation from EOs with a high percentage of alcohols and aldehydes.

**Figure 5:**
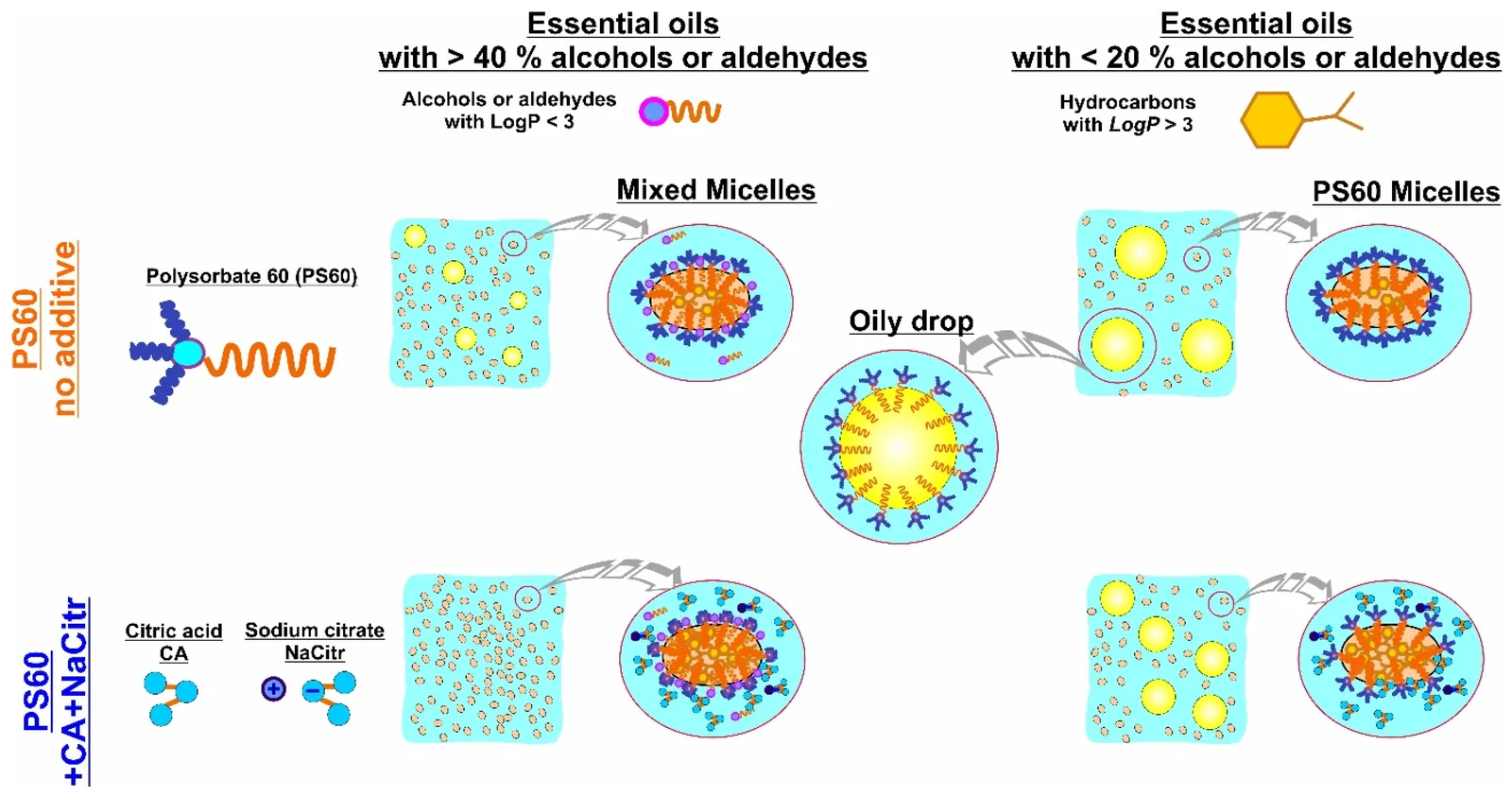
Schematic representation of emulsion types that are formed from essential oils with high (left part) and low (right part) contents of alcohols and aldehydes in a PS60 solution (first row) and PS60+CA+NaCitr (second row).

To test the hypothesis that a reduced shell thickness is the primary driving force behind the enhanced solubilization capacity of PS60 micelles in the presence of 40 wt % CA and 4.7 wt % NaCitr, control experiments were performed using 15 wt % PS60 solutions containing a much lower (0.7 M) concentration of either PG or the CA+NaCitr mixture. Previous structural characterizations indicated that at such lower concentrations of these additives, there is a sufficient amount of free water molecules available to fully hydrate the polyoxyethylene groups, maintaining a thick shell layer of 2.2 nm [35]. The resulting dispersions are shown in Figure S5. At this 0.7 M additive concentration, the only additives capable of ensuring complete microemulsion formation with 5 wt % TTO are KSorb and NaBenz; all other additives fail to solubilize the oil. Furthermore, LavO and MO, which form transparent microemulsions in the presence of 28.3 wt % PG or 40 wt % CA + 4.7 wt % NaCit, yield macroemulsions when the additive concentration is decreased to 0.7 M. Therefore, the increased shell thickness in the presence of a lower content of CA+NaCitr decreases the solubilization capacity of PS60 micelles with respect to hydrophobic components from TTO, LavO, and MO. The most probable explanation for this behavior is that highly hydrophobic hydrocarbon components (such as γ-terpinene) face a severe mass-transfer barrier when passing through a hydrated polyoxyethylene corona due to their low water solubility. When headgroup hydration is suppressed by high additive concentrations, this transport process becomes thermodynamically favorable, accelerating micellar incorporation and microemulsion formation (see Figure 5 below).

The interfacial tensions (IFTs) of four of the studied EOs (TTO, LavO, LemO, and OrO) were measured using the spinning drop method. The resulting interfacial tension values are shown in Table 3. OrO, which is almost entirely devoid of amphiphilic alcohols and aldehydes, exhibits the highest IFT against all tested surfactant solutions. This matches its inability to form a microemulsion under all studied conditions. In contrast, the presence of ≈20 mol % of aldehydes in LemO drives a significant reduction in IFT across all aqueous phases. Even the IFT against the PS60 solution drops sharply to 1.9 mN/m compared to 3.8 mN/m for OrO. Bearing in mind that the main hydrocarbon in both OrO and LemO is d-limonene, these findings demonstrate that the native aldehydes in LemO co-adsorb with PS60 molecules at the d-limonene/water interface and form a mixed surfactant–aldehyde adsorption layer. However, LemO still forms macroemulsions with all tested additives. This indicates that while a mixed interfacial monolayer lowers the IFT, it alone is insufficient to drive the solubilization of d-limonene in the PS60 micelles. As shown in Table 2, these aldehydes do not sufficiently dehydrate or compress the polyoxyethylene shell; therefore, d-limonene is unable to be transferred through the corona shell of the micelles from the aqueous phase into the micellar core.

The initial interfacial tensions of TTO and LavO against 15 wt % PS60 are nearly identical to the IFT of OrO (≈3.7 mN/m), indicating that the linalool/linalyl acetate in LavO and the terpinen-4-ol in TTO are relatively inefficient at displacing water or disrupting the pure surfactant monolayer on their own. However, introducing various additives into the aqueous phase (KSorb, PG, CA, or CA+NACitr) systematically lowers the IFT of the TTO systems. The interfacial tension decreases to ≈0.2 mN/m in the presence of 40 wt % CA + 4.7 wt % NaCitr, matching the near-instantaneous microemulsion equilibration observed visually in the bulk dispersions. For LavO, clear microemulsions are formed only when 15 wt % PS60 solutions containing either 28.3 wt % PG or 40 wt % CA + 4.7 wt % NaCit are used, where the IFT drops from 3.6 mN/m to 1.6 mN/m. For all other additives, the IFT remains above 2 mN/m, resulting in macroemulsion formation.

To establish a semi-quantitative framework that explains the significant difference in the solubilization capacity of PS60 micelles between TTO and EOs with low alcohol/aldehyde contents, we assumed that the LogP values of the individual components given in Table S1 provide a reliable estimate of their redistribution between the aqueous and micellar phases. As demonstrated in our previous work performed with more than 100 neutral non-ionic molecules, a linear correlation exists between micellar solubilization capacity and LogP for neutral molecules [38]. While recent studies [38,39,40] demonstrate that oil-phase-specific interactions dominate the partitioning of charged molecules, essential oil constituents consist almost exclusively of small, uncharged, lipophilic molecules (terpenes, terpenoids, alcohols, and aldehydes). For such neutral molecules, LogP serves as a good estimate for their partitioning between aqueous and micellar phases.

Note that the composition of the essential oils used is provided by their procedures, and only the composition of tea tree oil is taken from the literature under the assumption that the used TTO has a composition that is within the frame of international ISO standards (e.g., ISO 4730 for tea tree oil), which constrain constituent concentration ranges within relatively narrow specifications [41]. Note that the experimental data available in the literature for the composition of RO [42] and LavO [43,44] strongly agree with the composition given by the producer. By utilizing the octanol–water partition coefficient LogP of the individual EO components to model their redistribution between the aqueous and micellar phases, we estimate that if all of the 5 wt % EO is successfully incorporated into the 15 wt % PS60 + 40 wt % CA + 4.7 wt % NaCit micelles, the surfactant constitutes only ≈30 mol % of the total aggregate, while the remaining ≈70 mol % is dictated by the oil composition of EOs that form microemulsions (TTO, RO, LavO, and MO). For all EOs that do not form transparent microemulsions, the estimated cumulative percentage of these polar components drops below 15%, shifting the hydrocarbon fraction to above 60%, which could explain why these molecules cannot be fully solubilized in PS60 micelles. It should be mentioned that partition coefficients depend on the specific chemical structure of the hydrophobic phase [38,39,40]. Therefore, the micellar compositions provided in the current paper based on LogP values should be viewed as illustrative estimates rather than as experimentally determined compositions.

SAXS profiles show a direct decrease in micellar shell thickness upon adding citric acid and sodium citrate. Because each citric acid/citrate molecule binds approximately 8–12 water molecules, a 2.7 M additive concentration binds 21–32 M of available water. This leads to a significant decrease in the shell thickness of PS60 micelles because the amount of water molecules available to hydrate the ethoxy groups of the PS60 headgroup is limited. On the other hand, lower additive concentrations (0.7 M) increase the concentration of free water molecules, which are able to hydrate the ethoxy groups, leading to an increase in shell thickness.

Interfacial tension measurements support the localization of EO alcohols and aldehydes in the palisade layer of PS60 micelles. The interfacial tension of orange oil (predominantly d-limonene) against a PS60 solution is 3.8 mN/m, whereas for lemon oil (containing polar alcohols and aldehydes in addition to d-limonene), it is 1.9 mN/m. This two-fold decrease in interfacial tension indicates the inclusion of the polar constituents of lemon oil within the micellar interface.

Therefore, we can conclude that the presence of alcohols and aldehydes in the studied EOs can form a mixed-adsorption layer with PS60 molecules. However, for the efficient formation of microemulsions, along with low interfacial tension, the more hydrophilic components from EOs or additives from aqueous solutions must be able to decrease the micellar shell thickness through which the hydrophobic components have to be transferred into the core of the micelles (see Figure 5).

## 4. Materials and Methods

### 4.1. Materials

Polyoxyethylene sorbitan monostearate (polysorbate 60, TCI), denoted as PS60 in the text, is used as an emulsifier, and its concentration is fixed at 15 wt % in the aqueous phase. According to the manufacturer, PS60 has an HLB value of 14.9, which means that it will stabilize oil-in-water macroemulsions. According to the work of Abrar and Trathnigg [45], in addition to monoesters, PS60 also contains diesters, triesters, and tetraesters. As was shown in our previous study [35], the presence of these polyesters in the PS60 used leads to the formation of precipitates after 3 days of storage for solutions without additives.

The studied additives are citric acid (CA, Sigma-Aldrich, Taufkirchen, Germany), sodium benzoate (NaBenz, Sigma-Aldrich, Taufkirchen, Germany), potassium sorbate (KSorb, Teokom, Sofia, Bulgaria), sodium citrate (NaCitr, Na3C6H5O 5.5H2O, Teokom, Sofia, Bulgaria), and 1,2-propylene glycol (PG, Teokom, Sofia, Bulgaria). Deionized water purified by the Milli-Q Organex system (Millipore, Burlington, MA, USA) is used for solution preparation. The additives are dissolved in water, PS60 is added, and the mixture is stirred at 50 °C until complete dissolution. The concentrations of the additives in the solutions correspond to those in the systems with the best results obtained in the work by Ahtchi-Ali et al. [34], i.e., 10 wt % KSorb (0.7 M K-Sorb), 10 wt % NaBenz (0.7 M Na-Benz), 28.3 wt % PG (3.9 M PG), 20 wt % (1.1 M CA), or 40 wt %+4.7 wt % NaCitr (2.56 M CA+0.16 M Na-Citr) in the aqueous phase.

The concentrations of PS60 and additives studied here represent typical levels in concentrated flavor oil emulsions [34]. In industrial food applications, these concentrate premixes are diluted 100- to 1000-fold during subsequent production stages [46] to yield transparent soft drinks with the desired flavor intensity and preservative levels [47]. Moreover, this concentration of PS60 falls within the typical range employed in self-emulsifying drug delivery systems (SEDDS) to improve the oral delivery and absorption of hydrophobic drugs [48]. Additionally, PS60 solutions with the same additive concentration of 0.7 M are studied for comparison at the same water content.

Lavender, melissa, yarrow, rose, and chamomile essential oils are provided by Galen-N Ltd. (Sofia, Bulgaria); tea tree essential oil is provided by Rivana (Plovdiv, Bulgaria); and lemon and orange essential oils are provided by Citrus and Allied Essences Ltd. (Belcamp, MD, USA). The chemical composition of the oils was provided by the manufacturers via GC–MS analysis, summarized in Table S1, along with literature data for the solubility of each of the oil components and its LogP value. The summarized information from Table S1 is shown in Table 4.

Tea tree oil (TTO) contains ≈45% alicyclic alcohols, the main component of which is terpinen-4-ol (LogP ≈ 2.2), ≈50% alicyclic hydrocarbons (LogP ≈ 3.2), and ≈5% alicyclic ethers (LogP ≈ 2.5). Assuming that LogP values can provide a reasonable estimate for the distribution of TTO components between water and surfactant micelles, we can calculate that upon the addition of 1 wt % EO to the 15 wt % PS60 solution, ≈2% of TTO components will be molecularly soluble in water, whereas the other 98% will be incorporated into surfactant micelles. If we do not account for the redistribution of TTO components between water and surfactant micelles, the estimates show that ≈30% of TTO components can be dissolved in water when 1 wt % EO is used, and ≈12% of TTO components can be dissolved in water when 5 wt % EO is used for the preparation of a dispersion in the 15 wt % PS60 solution. This concentration decreases by almost two-fold when a solution of 15 wt % PS60+40 wt % CA+4.7 wt % NaCit is used due to the significantly lower water content in the latter solution, see Table 5.

Rose oil (RO) contains ≈70% aliphatic alcohols (citronellol, geraniol, and nerol) with LogP ≈ 2.9, and ≈25% long-chain aliphatic hydrocarbons with LogP ≈ 9.7. The most soluble substance in the RO used is ethanol, whose concentration is 0.71% of the RO. Because of its presence in the RO used, the estimated percentage of water-soluble components after accounting for redistribution of components between the surfactant and water is ≈3.4%, which is higher than that estimated for TTO components. If we do not account for the redistribution of RO components between water and surfactant micelles, then the estimates show that ≈32% of RO components can be dissolved in water when 1 wt % EO is used, and ≈12% of RO components can be dissolved in water when 5 wt % EO is used. It should be mentioned that the long-chain hydrocarbons present in RO have a much higher LogP than the LogP of alicyclic hydrocarbons in TTO, which is why we can expect that they will be less soluble in PS60 micelles than the components from TTO.

Lavender oil (LavO) contains ≈40% aliphatic alcohols (linalool), with LogP ≈ 2.6, and ≈40% aliphatic esters, with LogP ≈ 3.3. The estimate calculated under the assumption that the components are redistributed between surfactant micelles and water shows that only 0.8% of LavO components will be molecularly soluble in water when 1 wt % LavO is added to the 15 wt % PS60 solution. This percentage increases to 33% when 1% LavO is used and to 15% when 5% LavO is used, under the assumption that in both cases, the limiting solubility of different components in water is reached before their transfer into surfactant micelles.

Melisa oil (MO) contains ≈40% aliphatic aldehydes and ketones, with LogP ≈ 3.0, and ≈50% alicyclic hydrocarbons, with LogP ≈ 4.4. The estimated percentage of components that will remain soluble in the water phase after their redistribution with the surfactant is 0.2%, whereas the maximum soluble components from MO is 22% for the 1 wt % MO emulsion and 6% for 5 wt % MO. Chamomile oil (CO) contains ≈85% aliphatic esters, with LogP ≈ 3.0. The estimated percentage of components that will remain soluble in the water phase after their redistribution with the surfactant is 1.2%, whereas the maximum soluble components from CO are 20% for the 1 wt % EO emulsion and 8.4% for 5 wt % EO.

Yarrow oil (YO) contains ≈85% alicyclic hydrocarbons, with LogP ≈ 3.8. The estimated percentage of components that will remain soluble in the water phase after their redistribution with the surfactant is 0.3%, whereas the maximum soluble components from CO are 8.7% for the 1% EO emulsion and 6.3% for the 5% EO.

Lemon oil (LO) contains ≈80% alicyclic hydrocarbons, with LogP ≈ 3.3, and ≈20% aliphatic aldehydes and ketones, with LogP ≈ 2.8. The estimated percentage of components that will remain soluble in the water phase after their redistribution with the surfactant is 0.27%, whereas the maximum soluble components from LO are 8.2% for the 1% EO emulsion and 1.9% for the 5% EO.

Orange oil (OrO) contains 98% alicyclic hydrocarbons, with LogP ≈ 3.4. The estimated percentage of components that will remain soluble in the water phase after their redistribution with the surfactant is 0.12%, whereas the maximum soluble components from OrO are 0.45% for the 1% EO emulsion and 0.24% for the 5% EO.

### 4.2. Emulsion Preparation

To prepare 5 wt % EO emulsions, 14.25 g of the aqueous phase is placed in a glass vial, and 0.75 g of oil is added. To prepare 1 wt % oil emulsions, the amounts of the aqueous phase and oil are 14.85 g and 0.15 g, respectively. The emulsions are prepared at ambient temperature (23 ± 3 °C) by stirring with a magnetic stirrer (IKA) at 1000 rpm, using a magnetic stirrer bar with a diameter of 1 cm until transparency is achieved (but for no longer than 2 h). The prepared dispersions are kept in 20 mL glass bottles at ambient temperature (23 ± 3 °C). Next, we assessed the visual appearance of the emulsions, drop size, and stability over time. Pictures of the emulsions, taken 1 day after their preparation, are shown in this article.

It should be mentioned that the formation of a transparent dispersion under the hydrodynamic conditions used indicates the formation of a microemulsion rather than a nanoemulsion. Under the hydrodynamic conditions used, the achievable drop diameter by drop breakage induced by pressure fluctuation [49,50] is estimated to be ≈5 µm (assuming an energy dissipation rate of 10^3^ m^2^/s^3^, an interfacial tension of 0.2 mN/m, and a mass density of 10^3^ kg/m^3^). This estimate shows that the hydrodynamic conditions used are not capable of breaking drops with sizes smaller than 5 µm; as a consequence, the formed transparent dispersions cannot be nanoemulsions. Moreover, all formed transparent microemulsions (except those with RO, for which the gel phase formed on the top of the samples, without visible drops after 7 days of storage) are stable without any visual changes for at least 1 month when observed visually every 7 days. In the current study, the term “microemulsion” is used for transparent, macroscopically homogeneous systems containing the surfactant, oil, and water formed under mild mixing conditions. However, visual transparency over a one-month period does not necessarily mean that they are thermodynamically stable. Therefore, these systems are named microemulsions based on their formation and appearance rather than on their proven thermodynamic stability.

All transparent formulations with 1 wt % and 5 wt % EOs were prepared at least twice to assess formulation reproducibility and to determine the average time for oil solubilization in the studied solutions.

### 4.3. Drop Size Determination

The drop sizes of the transparent emulsions are determined via a Zetasizer Nano ZS (Malvern Instruments, Malvern, UK), set at a 173° scattering angle with a wavelength of 633 nm and a temperature of 25 °C without dilution. DLS measurements were performed on at least two independently prepared samples for each formulation, and each sample was measured at least three times. The mean volume diameter (*d*_VMEAN_) is used as a characteristic of the drop size in emulsions. The effect of the additives on the viscosity of the solutions is considered for the measurements. The values of *d*_VMEAN_ reported by Gazolu-Rusanova et al. [35] for the micelles are used for comparison. Note that transparent microemulsions prepared in a solution of 15 wt % PS60 + 2.56 M CA (40 wt %) + 0.16 M NaCitr (4.7 wt %) are diluted twice before measurement because of the high viscosity of the respective solutions.

Selected turbid emulsions are observed under polarized transmitted light using an optical Axioplan microscope (Zeiss, Oberkochen, Germany), equipped with a Zeiss Epiplan 50× objective, a digital camera, and a video recorder. The scale bar in the pictures included in the article is 20 μm.

### 4.4. Small-Angle X-Ray Scattering (SAXS) Analysis

SAXS measurements of the microemulsions are carried out on an in-house X-ray scattering system (XEUSS 3.0 SAXS/WAXS System, Xenocs, Sassenage, France), with a CuKα X-ray source operating at λ = 0.154 nm (Xeuss 3.0 UHR Dual source Mo/Cu, Xenocs, Sassenage, France) and an Eiger2 4M detector (Dectris Ltd., Baden Deattwil, Switzerland) with slit collimation. The sample-to-detector distance (SDD) of 1000 mm allowed a *q*-range of 0.01–0.5 Å^−1^. The data acquisition time is set to 20 min. Silver behenate is used as a standard to determine the SDD, and the coordinates of the beam center on the detector. Samples are enclosed in a vacuum-tight thin glass capillary with an outer diameter of 1 mm and a thickness of 10 µm. The scattered intensity is normalized to the incident intensity, corrected for the background scattering from the capillary, and calibrated to an absolute scale. The measurements are performed at ambient temperature (25 °C) after at least 3 months of storage for the transparent emulsions, or up to 1 week after preparation for the emulsions that are not stable for an extended period.

The analysis of the reduced SAXS data is performed using the SASView 6.2.0 software. The SAXS intensity has two contributions coming from the particle form factor P(q) and the interparticle interference factor S(q). The data are analyzed using different model functions (core-shell cylinder, ellipsoid, sphere, and microemulsion) implemented in SasView. The applied structure factor corresponds to hard-sphere interactions according to the Percus–Yevick equation for non-charged systems. The SLD values are determined via the NIST NCNR SLD calculator by accounting for the scattering length of each component and the volume it occupies [51]. In some cases, a combined model is shown to describe the experimental data.

The data from the work of Gazolu-Rusanova et al. [35] are used for comparison with the micellar solutions.

### 4.5. Interfacial Tension

The oil–water interfacial tension of lavender (ρ = 0.87 g/cm^3^ at 25 °C), tea tree (ρ = 0.877 g/cm^3^ at 25 °C), lemon (ρ = 0.865 g/cm^3^ at 25 °C), and orange (ρ = 0.84 g/cm^3^ at 25 °C) essential oils is measured at 25 ± 1 °C using a spinning drop tensiometer (SDT, Krüss GmbH, Hamburg, Germany). The profile of an oil drop in a bulk aqueous solution is fitted with the Laplace equation. The duration of each measurement is 10 min, and the value of the interfacial tension at the end of this time is used. Interfacial tensions are measured for at least three independently formed oil drops per oil phase in a given solution.

## 5. Conclusions

The solubilization capacity of 15 wt % polysorbate 60 (PS60) micelles, both without additives and when combined with various aqueous phase additives, was studied. Eight chemically diverse essential oils, which were classified into two groups based on their chemical composition, were studied. The first group consisted of essential oils with high contents of alicyclic or aliphatic alcohols or a high content of aliphatic aldehydes. This group included tea tree oil, rose oil, and lavender oil, which all contain more than 40% alcohols, as well as melissa oil, which contains 40% aldehydes. The second group comprised essential oils with low alcohol and aldehyde contents, specifically chamomile oil, yarrow oil, lemon oil, and orange oil, all containing less than 20% alcohols and aldehydes. The performance of these systems was tested in the presence of several additives, including 10 wt % potassium sorbate (KSorb), 10 wt % sodium benzoate (NaBenz), 20 wt % citric acid (CA), 28.3 wt % propylene glycol (PG), and a combination of 40 wt % CA and 4.7 wt % sodium citrate (CA+NaCitr).

The essential oils with low alcohol and aldehyde contents could not be efficiently solubilized in PS60 micelles, regardless of whether additives were present in the aqueous phase. Instead, they consistently formed macroemulsions characterized by micrometer-sized droplets. Among these, the smallest droplet sizes were obtained in the presence of the CA+NaCitr additive, which was shown to successfully lower the interfacial tension between the phases.

In contrast, the essential oils with high alcohol and aldehyde contents were efficiently solubilized in PS60 micelles in the presence of CA+NaCitr. This behavior is proposed to be driven by a cooperative mechanism. First, the alcohol and aldehyde components originating from the essential oils co-assemble with the PS60 molecules, forming mixed micelles. Second, the high concentration of CA+NaCitr dehydrates the ethoxy groups of the PS60 molecules. This dehydration shrinks the hydrophilic corona of the micelles, which significantly eases the mass transfer of the hydrophobic oil molecules into the micellar core.

When 10 wt % KSorb or NaBenz was used instead of 40 wt % CA+4.7 wt % NaCitr, the overall solubilization capacity of the PS60 micelles decreased. In the presence of these additives, microemulsions could only be formed using tea tree oil and rose oil, which possess the highest alcohol contents at over 45%. This weaker performance is proposed to be due to the higher water content of the KSorb and NaBenz solutions. The excess water increases the hydration of the PS60 ethoxy groups, creating a more robust hydrophilic barrier that restricts hydrophobic components from passing into the micelle core. This explanation is strongly supported by experiments conducted at lower concentrations of CA+NaCitr, where solubilization capacity similarly dropped due to increased ethoxy group hydration.

We propose that two primary requirements must be met for the efficient solubilization of the studied essential oils in PS60 micelles. The first requirement is the formation of mixed micelles between the surfactant molecules and the alcohols or aldehydes from the essential oils. This leads to the counterintuitive finding that transparent microemulsions are formed at intermediate essential oil concentrations, at which the essential oils supply enough alcohols or aldehydes to build the mixed micellar structure. The second requirement is the significant dehydration of the PS60 ethoxy groups to facilitate the transfer of the hydrophobic components of the studied essential oils. This leads to another unexpected result: reducing the concentration of water-soluble additives such as citric acid and sodium citrate actually impairs solubilization by allowing the micelle corona to rehydrate.

These underlying physical principles can serve as practical guidelines for formulating stable, transparent microemulsions across the cosmetics, food, and pharmaceutical industries.

## Figures and Tables

**Table 1:** Concentration range of EOs at which transparent microemulsions are formed after 30 min and after 24 h equilibration after EO addition to aqueous solution of 15 wt % PS60 + 40 wt % CA + 4.7 wt % NaCit.

EO	Concentration Range After 30 min, %	Concentration Range After 24 h, %
TTO	0.1–12.9%	0.1–12.9%
RO	3.4–12%	1–12%
LavO	4.9–6.2%	4.5–6.2%
MO	5.5–6.8%	4.0–6.8%
CO	<0.1%	<0.1%
YO	<0.1%	<0.1%
LemO	<0.1%	<0.1%
OrO	<0.1%	<0.1%

**Table 2:** The main characteristics of the micelles: core radii, *R*_xcore_, and *R*_ycore_; thickness of the shell, *t*_shell_; effective radius, *R*_eff_; and fitting error, χ_2_, as determined from the best fit of SAXS spectra by the core-shell ellipsoid model for measurements performed in 15 wt % PS60 and 15 wt % PS60+10 wt % KSorb in the presence of 5 wt % EOs. To fit the SAXS spectra, the SLD of the tails was fixed at 7.52 × 10^−6^ Å^−2^, the SLD of the shell was fixed at 10.4 × 10^−6^ Å^−2^, and the SLD of the solvent for the 15 wt % PS60 solution was fixed at 9.44 × 10^−6^ Å^−2^. For 15 wt % PS60+10 wt % KSorb, it was fixed at 9.68 × 10^−6^ Å^−2^, and for 15 wt % PS60+40 wt % CA+4.7 wt % NaCitr, it was fixed at 11.0 × 10^−6^ Å^−2^. The uniform shell thickness is assumed. The mean volume radius from DLS measurements is shown in the last column. The values in the rows with TTO* are calculated by using the estimated SLD value for the core of the micelles after accounting for the changed composition due to the solubilization of TTO molecules in the core of the micelles, which gives a value of 8.09 × 10^−6^ Å^−2^ instead of 7.52 × 10^−6^ Å^−2^.

15 wt % PS60	EO	*R*_xcore_, nm	*R*_ycore_, nm	*R*_eff_, nm	*t*_shell_, nm	φ	χ^2^	*R*_v_ from DLS, nm
No additives	TTO	4.8 ± 0.3	2.8 ± 0.3	6.4 ± 0.5	2.0 ± 0.3	0.25	1.3 × 10^−6^	Emulsion
	TTO*	5.0 ± 0.3	3.0 ± 0.3	6.4 ± 0.5	1.5 ± 0.3	0.25	1.1 × 10^−6^	Emulsion
	RO	4.3 ± 0.3	2.1 ± 0.2	5.5 ± 0.5	1.4 ± 0.2	0.19	2.0 × 10^−6^	Emulsion
+10 wt % KSorb	TTO	4.3 ± 0.3	2.4 ± 0.2	5.5 ± 0.5	1.6 ± 0.1	0.28	1.0 × 10^−6^	4.1 ± 0.1
	TTO*	4.3 ± 0.3	2.4 ± 0.2	5.5 ± 0.5	1.3 ± 0.1	0.28	1.2 × 10^−6^	4.1 ± 0.1
	RO	4.1 ± 0.2	2.1 ± 0.2	5.3 ± 0.5	1.2 ± 0.2	0.27	1.1 × 10^−6^	4.0 ± 0.3
+40 wt % CA + 4.7 wt % NaCitr	TTO	4.3 ± 0.4	2.4 ± 0.2	5.5 ± 0.4	0.6 ± 0.1	0.27	4.2 × 10^−6^	6.7 ± 0.3
	TTO*	4.3 ± 0.4	2.4 ± 0.2	5.5 ± 0.4	0.6 ± 0.1	0.27	4.2 × 10^−6^	6.7 ± 0.3
	RO	4.3 ± 0.4	2.2 ± 0.2	5.6 ± 0.4	0.8 ± 0.1	0.24	6.2 × 10^−6^	7.5 ± 0.6
	LavO	5.1 ± 0.4	3.1 ± 0.2	6.1 ± 0.4	0.2 ± 0.1	0.29	1.2 × 10^−5^	6.6 ± 0.5
	MO	4.8 ± 0.4	2.7 ± 0.2	5.8 ± 0.4	0.2 ± 0.1	0.26	7.9 × 10^−6^	9.8 ± 1.2
	CO	4.9 ± 0.4	3.0 ± 0.2	6.2 ± 0.4	1.1 ± 0.1	0.30	1.6 × 10^−5^	Emulsion
	YO	4.9 ± 0.4	3.1 ± 0.2	6.3 ± 0.4	0.7 ± 0.2	0.31	1.8 × 10^−5^	Emulsion
	LemO	4.5 ± 0.4	2.8 ± 0.2	6.1 ± 0.4	1.2 ± 0.2	0.31	2.8 × 10^−5^	Emulsion
	OrO	5.0 ± 0.4	3.2 ± 0.2	6.7 ± 0.4	1.0 ± 0.2	0.30	3.5 × 10^−5^	Emulsion

**Table 3:** Interfacial tensions in mN/m, measured after 10 min with a spinning drop tensiometer for EO drops placed in surfactant solutions with different compositions.

EO	15 wt % PS60	15 wt % PS60 + 10 wt % KSorb	15 wt PS60 + 28.3 wt % PG	15 wt PS60 + 20 wt % CA	15 wt PS60 + 40 wt % CA + 4.7 wt % NaCitr
TTO	3.7 ± 0.1	0.7 ± 0.1	1.5 ± 0.1	0.6 ± 0.1	0.2 ± 0.1
LavO	3.6 ± 0.1	2.3 ± 0.1	1.6 ± 0.1	2.2 ± 0.1	1.6 ± 0.1
LemO	1.9 ± 0.1	0.8 ± 0.1	1.1 ± 0.1	1.2 ± 0.1	1.0 ± 0.1
OrO	3.8 ± 0.1	3.0 ± 0.1	2.4 ± 0.1	3.1 ± 0.1	2.6 ± 0.1

**Table 4:** The molar fractions of the main components in the studied essential oils.

EO	TTO	RO	LavO	MO	CO	YO	LemO	OrO
**Alicyclic** **alcohols**	45% LogP ≈ 2.2 Terpinenol					3% LogP ≈ 2.2 1-Terpinen-4-ol		
**Aliphatic** **alcohols**		75% LogP ≈ 2.9 Citronellol Geraniol	40% LogP ≈ 2.6 Linalool		3% LogP ≈ 1.8			
**Aldehydes and ketones**				40% LogP ≈ 3.0 Geranial	5% LogP ≈ 2.2	5% LogP ≈ 3.1	18% LogP ≈ 2.8 C8Aldehyde	
**Esters and Ethers**	5% LogP ≈ 2.5		40% esters LogP ≈ 3.3 Linalyl acetate		85% LogP ≈ 3.0 Butyl 3-methyl- 2-butenoate	3.5% ethers 1.5% esters LogP ≈ 3.0	2% LogP ≈ 3.5	
**Alicyclic** **hydrocarbons**	50% LogP ≈ 3.2 γ-Terpinene		5% LogP ≈ 3.8	53% LogP ≈ 4.4 β-Caryophyllene	7% LogP ≈ 3.7	85% LogP ≈ 3.8 β-pinene	80% LogP ≈ 3.3 d-limonene	98% LogP ≈ 3.4 d-limonene
**Aliphatic** **hydrocarbons**		25% LogP ≈ 9.7 n-paraffins C17-C23	15% LogP ≈ 4.4	7% LogP ≈ 4.3		2% LogP ≈ 4.4		2% LogP ≈ 4.3

**Table 5:** Water solubility for dispersions containing 1 wt % EO or 5 wt % EO in 15 wt % PS60 or 15 wt % PS60 + 40 wt % CA + 2.7 wt % NaCitr, under the assumption that the limiting solubility of each component is reached. The values in parentheses for water solubility are calculated after accounting for their redistribution between the aqueous phase and pseudo-micellar phase in agreement with their LogP values. Sources and data used for estimations are provided in the Supplementary Information.

EO	TTO	RO	LavO	MO	CO	YO	LemO	OrO
**1 wt % EO** **15 wt % PS60**	30% (1.9%)	32% (3.4%)	33% (0.8%)	22% (0.3%)	20% (1.2%)	8.8% (0.3%)	8.1% (0.3%)	0.5% (0.1%)
**5 wt % EO** **15 wt % PS60**	12% (1.9%)	12% (3.4%)	15% (0.8%)	6.2% (0.3%)	8.4% (1.2%)	6.3% (0.3%)	1.9% (0.1%)	0.2% (0.03%)
**5 wt % EO** **15 wt % PS60 +**	6.7% (0.9%)	9.2% (2.7%)	9.9% (0.4%)	3.4% (0.2%)	4.3% (0.5%)	5.6% (0.1%)	0.9% (0.04%)	0.2% (0.01%)
**40 wt % CA** **+4.7 wt % NaCitr**								

## Data Availability

Data will be available upon request.

## Supplementary Materials

The following supporting information can be downloaded at: https://www.mdpi.com/article/10.3390/ijms27188165/s1. References [32,52,53] are cited in the supplementary materials.

## Abbreviations

The following abbreviations are used in this manuscript:

TTO	Tea tree oil
RO	Rose oil
LavO	Lavender oil
MO	Melissa oil
CO	Chamomile oil
YO	Yarrow oil
LemO	Lemon oil
OrO	Orange oil
EO	Essential oil
PS60	Polysorbate 60
KSorb	Potassium sorbate
NaBenz	Sodium benzoate
CA	Citric acid
NaCitr	Sodium citrate
SAXS	Small-angle X-ray scattering
SLD	Scattering length density
