# Supplementary material for: Microemulsions of Essential Oils: Characterization and Phase Behavior
Source: Int J Mol Sci. 2026 Sep 14;27(18):8165. doi: 10.3390/ijms27188165 (PMC13607099; doi:10.3390/ijms27188165)
Supplement: Supplementary file 1 [file ijms-27-08165-s001.zip › ijms-4501335-supplementary.pdf]

Supplementary Information

Microemulsions of Essential Oils:

Characterization and Phase Behavior

Dilek Gazolu-Rusanova[a,b], Zlatina Mitrinova[a], Ivan Lesov[a], Slavka Tcholakova[a]*

[a] *Department of Chemical and Pharmaceutical Engineering, Faculty of Chemistry and Pharmacy, Sofia University, 1164, Sofia, Bulgaria*
[b] *Centre of Competence "Sustainable Utilization of Bio-resources and Waste of Medicinal and Aromatic Plants for Innovative Bioactive Products" (BIORESOURCES BG), Sofia, Bulgaria*

*__Corresponding author__:
Prof. Slavka Tcholakova
Department of Chemical and Pharmaceutical Engineering
Faculty of Chemistry and Pharmacy, Sofia University
1 James Bourchier Ave., 1164 Sofia
Bulgaria

Phone: (+359-2) 962 5310
Fax: (+359-2) 962 5643
E-mail: SC@LCPE.UNI-SOFIA.BG

Table S1. Chemical composition and estimated solubility of the studied oils. The main components are highlighted.

(A) Tea tree oil – composition reported in Carson et al., 2006.

**Compound**	**Content, %**	**Molecular mass, g/mol**	**Solubility in water at 20-25 °C, g/L**	**LogP**
Terpinen-4-ol	40.1	154.25	2.5	2.2
γ-Terpinene	23	136.23	0.005915	2.8
α-Terpinene	10.4	136.23	0.005915	2.8
1,8-Cineole(eucalyptol)	5.1	154.25	3.5	2.5
Terpinolene	3.1	136.23	0.0095	2.8
α-Terpineol	2.9	154.25	2.42	1.8
α-Pinene	2.6	136.23	0.00249	2.8
ρ-Cymene	2.4	134.22	0.023	4.1
Aromadendrene	1.5	204.35	0.00007057	4.7
δ-Cadinene	1.3	204.35	0.001	3.8
Limonene	1	136.23	0.0138	3.4
Globulol	0.2	222.37	0.001	3.7
Sabinene	0.2	136.23	0.00249	3.1
Virirdiflolol	0.1	222.37	0.001	3.7

(B) Rose oil

**Compound**	**Content, %**	**Molecular mass, g/mol**	**Solubility in water at 20-25 °C, g/L**	**LogP**
Citronellol	29.45	156.26	0.31	3.2
Geraniol	18.82	154.25	0.686	2.9
Nerol	10.24	154.25	1.311	2.9
Nonadecane C19	8.94	268.5	0.00001	9.9
Nonadecene C19	4.22	268.5	0.00001	9.9
Heneicosane C21	3.86	296.6	0.00001	11
Heptadecane C17	1.13	240.5	0.00001	8.8
Tricosane C23	1.04	324.6	0.00001	12.1
Eicosane C20	0.20	282.5	0.00001	10.4
Farnesole	1.95	222.37	0.001287	4.8
Methyl eugenol	1.12	178.23	0.5	2.5
Eugenol	0.98	164.20	2.46	2.0
Geranyl acetate	0.92	196.29	0.058	3.5
Linalool	0.84	154.25	1.589	2.7
Ethanol	0.71	46.07	1000	-0.1
Phenylethyl alcohol	0.65	122.16	16	1.4
Cis-Rose oxide	0.25	154.25	0.06397	2.9
Trans-Rose oxide	0.11	154.25	0.06397	2.9

(C) Lavender oil

Compound	Content, %	Molecular mass, g/mol	Solubility in water at 20-25 °C, g/L	LogP
Linalool	27.18	154.25	1.589	2.7
Linalyl acetate	31.08	196.29	0.061	3.3
Cis-β-ocimeme	5.60	136.23	0.170	4.3
Farnesene	5.05	204.35	0.0002	6.1
Caryophyllene	4.72	204.35	0.00005	4.4
Terpinen-4-ol	4.00	154.25	2.500	2.2
Lavandulol acetate	3.18	196.29	0.01644	3.6
Trans-β-ocimene	2.34	136.23	0.170	4.3
Lavandulol	1.20	154.25	0.79366	3.0
3-Octanone	1.04	128.21	2.600	2.3
α -terpineol	0.89	154.25	2.420	1.8
1-Octen-3-yl-acetate	0.86	170.25	0.05158	3.1
Borneol	0.65	154.25	0.740	2.7
Eucalyptol	0.61	154.25	3.500	2.5
Myrcene	0.56	136.23	0.0056	4.3
Hexyl butyrate	0.34	172.26	0.045656	3.3
Limonene	0.32	136.23	0.013800	3.4
β-Phellandrene	0.32	136.23	0.002452	3.4
1-Octen-3-ol<br>Camphor	0.32<br>0.26	128.21<br>152.23	1.8360<br>1.2000	2.6<br>2.2
α-Pinene	0.20	136.23	0.00249	2.8
Camphene	0.15	136.23	0.0046	3.3
β-Pinene	0.04	136.23	0.00249	3.1

(D) Melissa oil

Compound	Content, %	Molecular mass, g/mol	Solubility in water at 20-25 °C, g/L	LogP
β-Caryophyllene	23.59	204.35	0.003	4.4
Geranial	14.19	152.23	0.59	3.0
Germacrene D	10.65	204.35	0.0022	4.7
Neral	9.54	152.23	0.59	3.0
Citronellal	5.17	154.25	0.06	3.0
Cis-β-ocimeme	3.53	136.23	0.17	4.3
D-limonene	3.52	136.23	0.0138	3.4
Caryophyllene oxide	0.82	220.35	0.004626	3.6
Citronelool	0.72	156.26	0.31	3.2
Linalool	0.35	154.25	1.589	2.7
Trans-β-ocimeme	0.33	136.23	0.17	4.3
6-methyl-5-heptene-2-one	0.3	126.20	3.54	1.9
β-Myrcene	0.28	136.23	0.00109	4.3
1-Octen-3-ol	0.16	128.21	1.836	2.6

(E) Chamomile oil (roman)

Compound	Content, %	Molecular mass, g/mol	Solubility in water at 20-25 °C, g/L	LogP
Butyl 3-methyl-2-butenoate	33.14	156.22	0.51838	3.2
Isopentyl 3-methyl-2-butenoate<br>pentyl 3-methyl-2-butenoate	24.02	170.25	0.0539	3.2
Allyl-3-methyl-2-butenoate	8.87	140.18	0.0539	2.2
Pinocarvone	4.74	150.22	0.1172	2.2
Isoamyl isobutyrate	3.85	158.24	0.1361	2.8
Limonene	3.76	136.23	0.0138	3.4
Pinocarveol	3.13	152.23	0.9581	1.8
Isobutyl isobutanoate	2.99	144.21	1	2.5
Isobutyl methacrylate	1.89	142.20	1.3	2.7
Germacrene	1.72	204.35	0.0013	4.7
Butyl 2-methylbutanoate	0.87	158.24	0.1178	2.8
Amyl methacrylate	0.75	156.22	0.1219	3.4
Hexyl isobutyrate	0.41	172.26	0.9581	3.5
α-Pinene	0.38	136.23	0.00249	2.8
β-Pinene	0.35	136.23	0.00249	3.1
Camphene	0.1	136.23	0.0046	3.3

(F) Yarrow oil

Compound	Content, %	Molecular mass, g/mol	Solubility in water at 20-25 °C, g/L	LogP
β-pinene + sabinene	28.57	136.23	0.00249	3.1
Chamazulene	11.93	184.28	0.0002979	4.4
β-Caryophyllene	11.64	204.35	0.001	4.4
Germacrene D	9.38	204.35	0.0022	4.7
Artemisia ketone	3.9	152.23	0.172	3.1
1-Terpinen-4-ol	2.1	154.25	2.5	2.2
α-pinene	2.01	136.23	0.00249	2.8
α -Caryophyllene/α-Humulene	1.95	204.35	0.001	4.5
1,8-Cineole	1.85	154.25	3.5	2.5
Limonene	1.69	136.23	0.0138	3.4
Bornyl acetate	1.19	196.29	0.01	3.3
Cymene	0.95	134.22	0.023	4.1
Caryophyllene oxide	0.91	220.35	0.004626	3.6
β-Myrcene	0.86	136.23	0.00109	4.3
α-Terpinene	0.68	136.23	0.005915	2.8
Terpinolene	0.37	136.23	0.0095	2.8
alfa-Thujene	0.31	136.23	0.16	2.8
Camphor	0.25	152.23	1.2	2.2
Camphene	0.23	136.23	0.0046	3.3
β-Phellandrene	0.21	136.23	0.002452	3.4
Santolina triene	0.13	136.23	0.00249	4.2

(G) Lemon oil

Compound	Content, %	Molecular mass, g/mol	Solubility in water at 20-25 °C, g/L	LogP
D-limonene	56.3	136.23	0.0138	3.4
γ-terpinolene	13.94	136.23	0.0095	2.8
Aldehyde C-8	9.9	128.21	0.00001	2.7
Geranial	5.22	152.23	0.59	3.0
β-pinene	5.03	136.23	0.00249	3.1
Neral	3.28	152.23	0.59	3.0
β-bisabolene	2.17	204.35	0.00001	5.2
Neryl acetate	1.39	196.29	0.0125	3.5
Geranyl acetate	1.1	196.29	0.0125	3.5
β-caryophyllene	0.86	204.35	0.00005	4.4
ρ-Cymene	0.19	134.22	0.023	4.1

(H) Orange oil

Compound	Content, %	Molecular mass, g/mol	Solubility in water at 20-25 °C, g/L	LogP
D-limonene	96.33	136.23	0.0138	3.4
Myrcene/Octanal	1.66	136.23	0.0056	4.3
α-pinene	0.341	136.23	0.00249	2.8
Linalool	0.175	154.25	1.589	2.7
ρ-Cymene	0.09	134.22	0.023	4.1
Aldehyde C10	0.072	156.26	0.0001	3.8
Limonene oxides	0.02	152.23	0.1372	2.5

References:

The information about the solubility, molecular mass and LogP of the components is taken from the web sites:
https://pubchem.ncbi.nlm.nih.gov
http://www.thegoodscentscompany.com;
http://www.chemicalland21.com
and the articles of Rao & McClements [32] and Fichan, Larroche, & Gros (1999).
I. Fichan, C. Larroche, and J. B. Gros (1999) Water Solubility, Vapor Pressure, and Activity Coefficients of Terpenes and Terpenoids, *J. Chem. Eng. Data*, *44* (1), 56-62. DOI: 10.1021/je980070+
The information about the composition of the tea tree oil was taken from:
Carson, C.F., Hammer, K.A., Riley, T.V. (2006) Melaleuca alternifolia (Tea Tree) Oil: a Review of Antimicrobial and Other Medicinal Properties, *Clinical microbiology reviews*, *19*, 1, 50–62

Table S2. The time required for the emulsion to become transparent for 1 % Tea tree and 1 % Rose oil in a solution of 15 % Polysorbate 60 ± additive. Emulsions are prepared at ambient conditions.

**EO**	**Oil content**	**Concentration of additive in aqueous phase**	**Time required to achieve a transparent emulsion**
Tea tree	1 %	No additive	15 min
		10 wt% K-sorbate	5 min
		40 wt% Citric acid + 4.7 wt% Na-citrate	< 1 min
Rose	1 %	40 wt% Citric acid + 4.7 wt% Na-citrate	20 h

Table S3. The main characteristics of the micelles: core radii, R_{xcore} and R_{ycore}; thickness of the shell, t_{shell}: effective radius, R_{eff} and fitting error, χ^2 as determined from the best fit of SAXS spectra by core-shell ellipsoid model for measurements performed in 15 wt % PS60 and 15 wt % PS60+10 wt % KSorb without and in presence of 1 wt % EO. For fitting the SAXS spectra the SLD of the tails was fixed to 7.52×10^{-6} Å^{-2}, SLD of the shell was fixed at 10.4×10^{-6} Å^{-2}, SLD of the solvent for 15 wt % PS60 solution was fixed to 9.44×10^{-6} Å^{-2} and for 15 wt % PS60+10 wt % KSorb was fixed to 9.68×10^{-6} Å^{-2}. Uniform shell thickness is assumed. The mean volume radius from DLS measurements is shown in the last column.

15 wt % PS60	EO		Parameters from SAXS						R_v from DLS, nm
	Type	wt%	R_{xcore},nm	R_{ycore},nm	R_{eff}, nm	t_{shell}, nm	φ	χ^2	
No additive	No	0	2.1±0.1	3.4±0.2	5.2±0.5	1.8±0.2	0.21	2.1×10^{-7}	2.9±0.1
	TTO	1	2.2±0.2	3.7±0.2	4.9±0.3	1.8±0.2	0.22	1.7×10^{-7}	3.0±0.2
	RO	1	2.1±0.1	3.9±0.2	4.8±0.2	1.8±0.2	0.22	1.4×10^{-7}	Emulsion
	MO	1	2.2±0.1	4.2±0.4	4.6±0.3	1.8±0.2	0.18	8.3×10^{-8}	Emulsion
+10 wt % KSorb	No	0	1.9±0.1	2.9±0.2	4.1±0.2	1.7±0.1	0.20	3.3×10^{-7}	2.7±0.2
	TTO	1	2.1±0.1	3.7±0.2	4.5±0.3	1.7±0.1	0.25	2.7×10^{-7}	3.3±0.4
	RO	1	2.0±0.1	4.5±0.3	4.6±0.5	2.0±0.2	0.24	1.3×10^{-7}	Emulsion
	MO	1	2.0±0.1	4.1±0.2	4.5±0.4	1.9±0.2	0.25	1.4×10^{-7}	Emulsion

Table S4. Time required for formation of transparent microemulsions for 5 % essential oil in solution of 15 % Polysorbate 60 ± additives. Emulsions are prepared at ambient conditions.

EO	Oil content	Concentration of additive in aqueous phase	Time required to achieve a transparent emulsion
Lavender	5 %	28.3 wt% Propylene glycol	15 min
		40 wt% Citric acid + 4.7 wt% Na-citrate	17 min
Melissa	5 %	40 wt% Citric acid + 4.7 wt% Na-citrate	13 min
Rose	5 %	28.3 wt% Propylene glycol	< 1 min
		10 wt% Na-benzoate	55 min
		10 wt% K-sorbate	11 min
		20 wt% citric acid	12 min
		40 wt% Citric acid + 4.7 wt% Na-citrate	< 1 min
Tea tree	5 %	0.7 M Propylene glycol	~ 1 week storage
		28.3 wt% Propylene glycol (3.9 M)	2 min
		10 wt% Na-benzoate	1 h 50 min
		10 wt% K-sorbate	15 min
		0.7 M citric acid	1 night storage
		20 wt% citric acid (1.1 M)	55 min
		0.7 M Citric acid + Na-citrate	~1 month
		40 wt% Citric acid + 4.7 wt% Na-citrate (2.7 M)	2 min

Table S5. The main characteristics of the micelles: core radii, R_{xcore} and R_{ycore}; thickness of the shell, t_{shell}: effective radius, R_{eff} and fitting error, χ^2 as determined from the best fit of SAXS spectra by core-shell ellipsoid model for measurements performed in 15 wt % PS60 and 15 wt % PS60+10 wt % KSorb without and in presence of 1 wt % EO. For fitting the SAXS spectra the SLD of the tails was fixed to 7.52×10^{-6} Å^{-2}, SLD of the shell was fixed at 10.4×10^{-6} Å^{-2}, SLD of the solvent for 15 wt % PS60 solution was fixed to 9.44×10^{-6} Å^{-2} and for 15 wt % PS60+10 wt % KSorb was fixed to 9.68×10^{-6} Å^{-2}. The uniform shell thickness is assumed.

15 wt % PS60	EO	wt % EO	Parameters from SAXS						
			Used model	R_{xcore}, nm	R_{ycore}, nm	R_{eff}, nm	t_{shell}, nm	φ	χ^2
No Additive	No	0	Prolate	2.1±0.1	3.4±0.2	5.2±0.5	1.8±0.2	0.21	2.1×10^{-7}
			Oblate	2.9±0.2	1.8±0.1	4.7±0.5	1.8±0.2	0.21	2.5×10^{-7}
	TTO	1	Prolate	2.2±0.2	3.7±0.2	4.9±0.3	1.8±0.2	0.22	1.7×10^{-7}
			Oblate	3.1±0.2	1.8±0.1	4.7±0.5	1.8±0.2	0.22	1.5×10^{-7}
		5	Prolate	3.5±0.3	6.2±0.8	6.4±0.5	2.1±0.3	0.27	3.3×10^{-6}
			Oblate	4.8±0.3	2.8±0.3	6.4±0.5	2.0±0.3	0.25	1.3×10^{-6}
	RO	1	Prolate	2.1±0.1	3.9±0.2	4.8±0.2	1.8±0.2	0.22	1.4×10^{-7}
			Oblate	3.1±0.2	1.7±0.1	4.5±0.3	1.6±0.2	0.22	1.3×10^{-7}
		5	Prolate	2.8±0.2	5.5±0.3	5.5±0.5	1.5±0.2	0.19	4.9×10^{-6}
			Oblate	4.3±0.3	2.1±0.2	5.5±0.5	1.4±0.2	0.19	2.0×10^{-6}
	MO	1	Prolate	2.2±0.1	4.2±0.4	4.6±0.3	1.8±0.2	0.18	8.3×10^{-8}
			Oblate	3.2±0.3	1.8±0.2	4.7±0.3	1.8±0.2	0.19	1.4×10^{-7}
+10 wt % KSorb	No	0	Prolate	1.9±0.1	2.9±0.2	4.1±0.2	1.7±0.1	0.20	3.3×10^{-7}
			Oblate	2.7±0.3	1.5±0.2	4.3±0.2	1.5±0.1	0.15	3.9×10^{-7}
	TTO	1	Prolate	2.1±0.1	3.7±0.2	4.5±0.3	1.7±0.1	0.25	2.7×10^{-7}
			Oblate	2.9±0.2	1.7±0.1	4.4±0.3	1.7±0.1	0.25	2.9×10^{-7}
		5	Prolate	2.9±0.2	5.6±0.3	5.5±0.5	1.7±0.1	0.28	2.2×10^{-6}
			Oblate	4.3±0.3	2.3±0.2	5.5±0.5	1.6±0.1	0.28	1.0×10^{-6}

	RO	1	Prolate	2.0±0.1	4.5±0.3	4.6±0.5	2.0±0.2	0.24	1.3×10^{-7}
			Oblate	2.9±0.3	1.7±0.1	4.4±0.3	1.6±0.2	0.26	2.5×10^{-7}
		5	Prolate	2.8±0.2	5.1±0.5	5.3±0.5	1.2±0.2	0.27	2.8×10^{-6}
			Oblate	4.1±0.2	2.1±0.2	5.3±0.5	1.2±0.2	0.27	1.1×10^{-6}
	MO	1	Prolate	2.0±0.1	4.1±0.2	4.5±0.4	1.9±0.2	0.25	1.4×10^{-7}
			Oblate	2.9±0.3	1.7±0.1	4.4±0.4	1.6±0.2	0.26	2.0×10^{-7}
+40 wt % CA+ 4.7 wt % NaCitr	TTO	5	Prolate	2.9±0.1	5.1±0.5	5.5±0.4	1.0±0.2	0.28	1.3×10^{-5}
			Oblate	4.3±0.4	2.4±0.2	5.5±0.4	0.6±0.1	0.27	4.2×10^{-6}
	RO	5	Prolate	3.1±0.2	5.8±0.5	5.6±0.4	0.2±0.2	0.24	3.3×10^{-5}
			Oblate	4.3±0.4	2.2±0.2	5.6±0.4	0.8±0.1	0.24	6.2×10^{-6}
	LavO	5	Prolate	3.5±0.2	6.0±0.5	6.1±0.4	0.7±0.2	0.29	5.0×10^{-5}
			Oblate	5.1±0.4	3.1±0.2	6.1±0.4	0.2±0.1	0.29	1.2×10^{-5}
	MO	5	Prolate	3.3±0.2	5.9±0.5	5.8±0.4	0.8±0.2	0.27	3.5×10^{-5}
			Oblate	4.8±0.4	2.7±0.2	5.8±0.4	0.2±0.1	0.26	7.9×10^{-6}
	CO	5	Prolate	3.6±0.2	6.0±0.5	6.2±0.4	0.2±0.2	0.30	2.0×10^{-5}
			Oblate	4.9±0.4	3.0±0.2	6.2±0.4	1.1±0.1	0.30	1.6×10^{-5}
	YO	5	Prolate	3.7±0.2	5.9±0.5	6.3±0.4	0.8±0.2	0.31	2.9×10^{-5}
			Oblate	4.9±0.4	3.1±0.2	6.3±0.4	0.7±0.2	0.31	1.8×10^{-5}
	LemO	5	Prolate	3.4±0.2	5.4±0.5	6.1±0.4	0.9±0.2	0.31	3.0×10^{-5}
			Oblate	4.5±0.4	2.8±0.2	6.1±0.4	1.2±0.2	0.31	2.8×10^{-5}
	OrO	5	Prolate	3.8±0.2	6.3±0.5	6.7±0.4	1.4±0.2	0.30	3.6×10^{-5}
			Oblate	5.0±0.4	3.2±0.2	6.7±0.4	1.0±0.2	0.30	3.5×10^{-5}

Figure S1. Microscope images of drops formed in emulsions prepared by using 1 wt % EO and 15 wt % PS60; 15 wt % PS60 + 10 wt % KSorb; 15 wt % PS60 + 40 wt % CA + 4.7 wt % NaCitr. The distance between the bars is 20 μm.

EO	1 wt % EO + 15 wt % PS60		
	No Additive	+ 10 wt % KSorb	+40 wt %CA+4.7 wt % NaCitr
MO	-	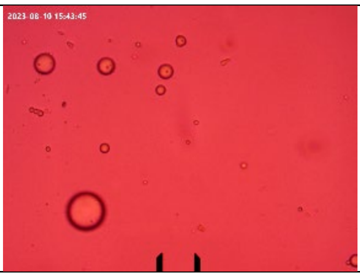	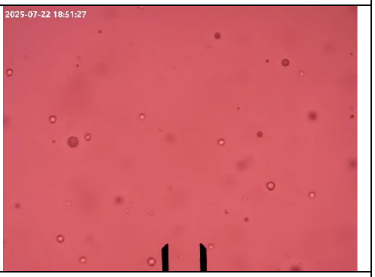
CO	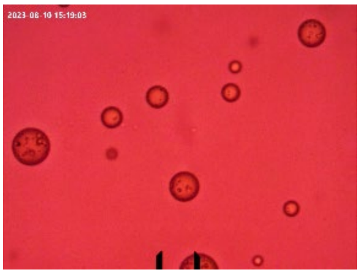	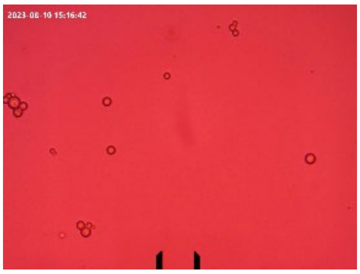	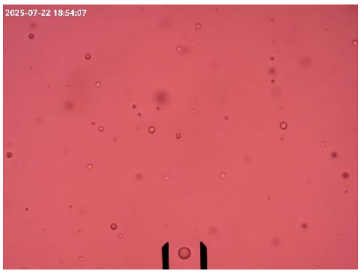
YO	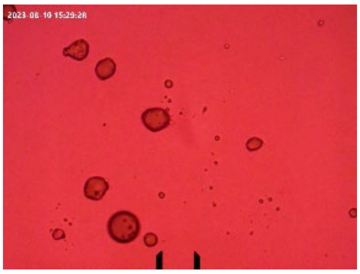	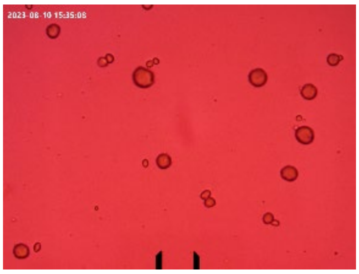	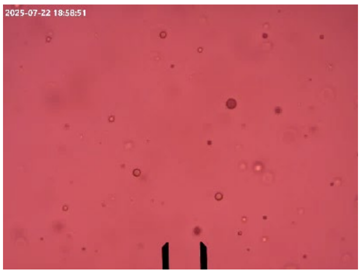
OrO	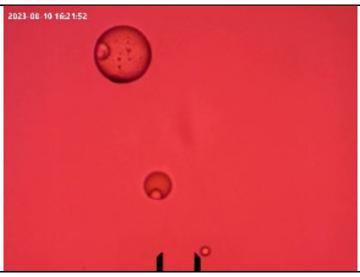	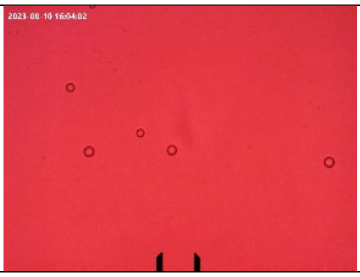	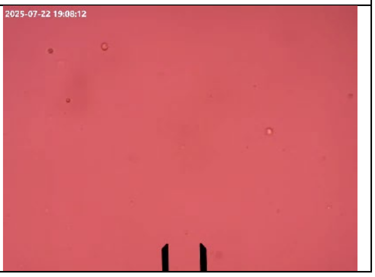

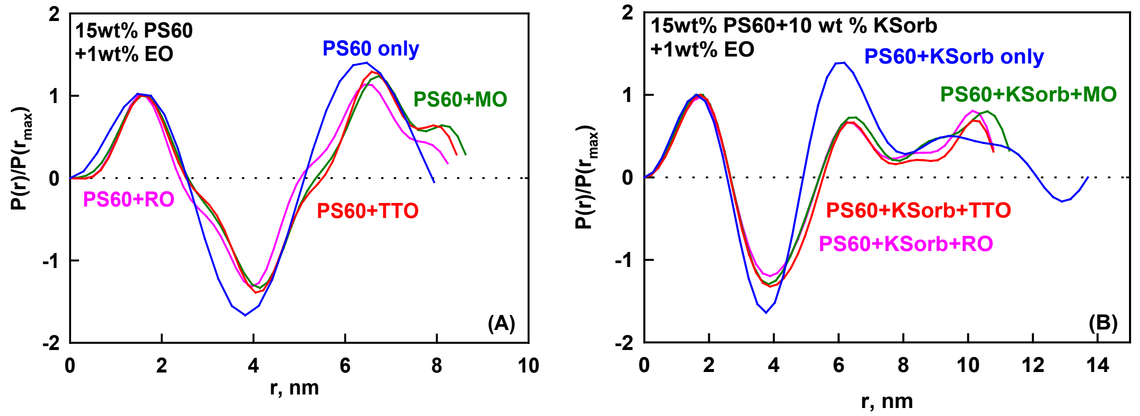

Figure S2. Normalized pair distance distribution function of dispersions of 1 wt % EO in (A) 15 wt % PS 60; (B) 15 wt % PS 60 + 10 wt % KSorb for TTO (red curves); RO (pink curves); MO (green curves) and for solutions only (blue curves).

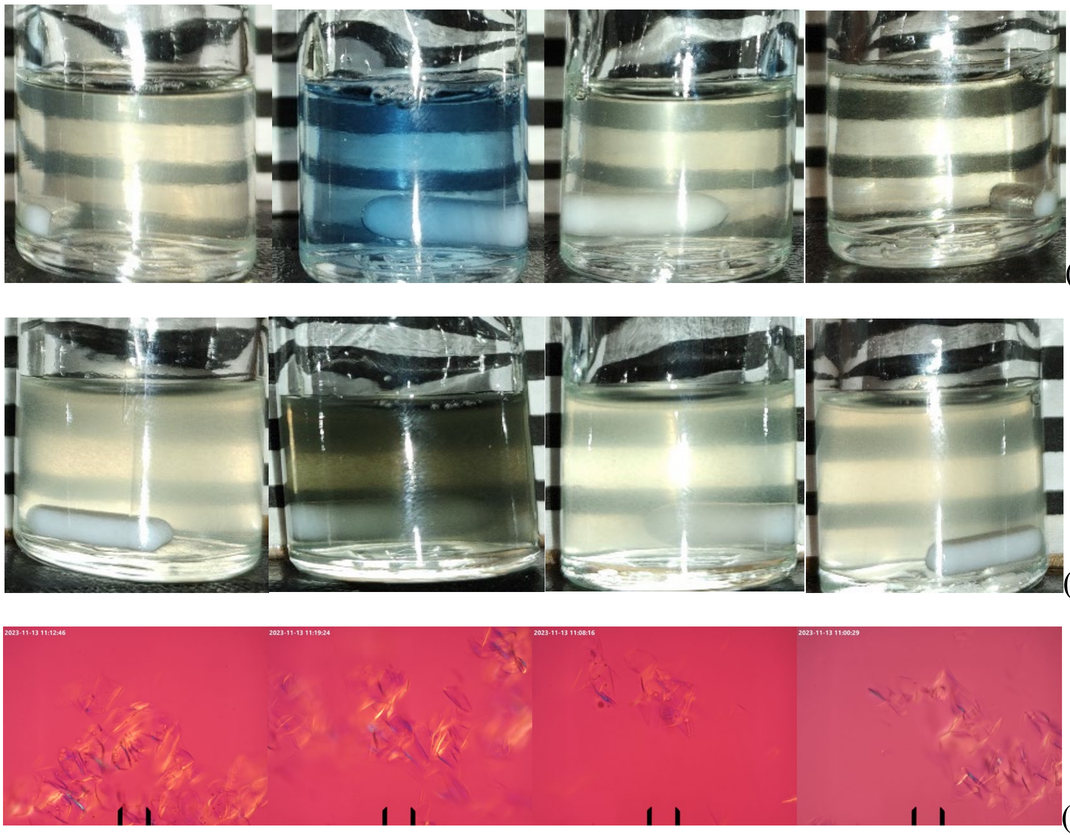

Figure S3. Dispersions formed with 0.1 wt% EOs and 15 wt % PS60 + 40 wt % CA + 4.7 wt % NaCit after (A) their preparation and (B) after 3 days of storage. (C) Microscope images taken from the emulsions stored 3 days. The distance between the bars is 20 μm.

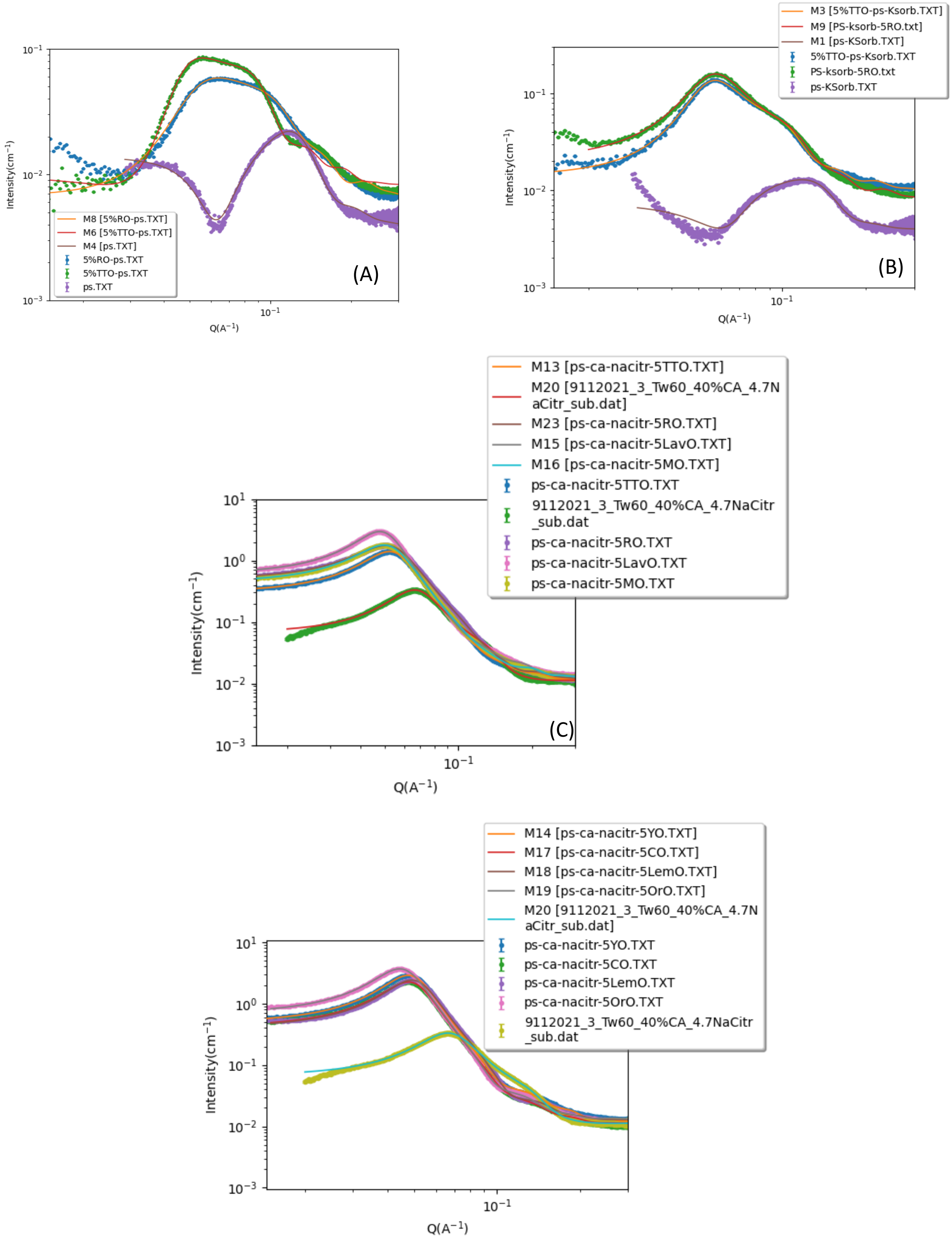

Figure S4. SAXS spectra and best fit with parameters given in Table S4 for (A) 15 wt % PS60 without and with 5 wt % EOs; (B) 15 wt % PS60 + 10 wt % KSorb without and with 5 wt % EOs; (C) 15 wt % PS60 +40 wt % CA+4.7 wt % NaCitr without and with 5 wt % EOs for EOs that form microemulsions and (D) 15 wt % PS60 +40 wt % CA+4.7 wt % NaCitr without and with 5 wt % EOs for EOs that form macroemulsions. The fits for solutions without EOs are performed with prolate ellipsoids, whereas those with 5 wt % EOs are performed with oblate ellipsoids.

No additive **PG** **KSorb** **CA+NaCitr**

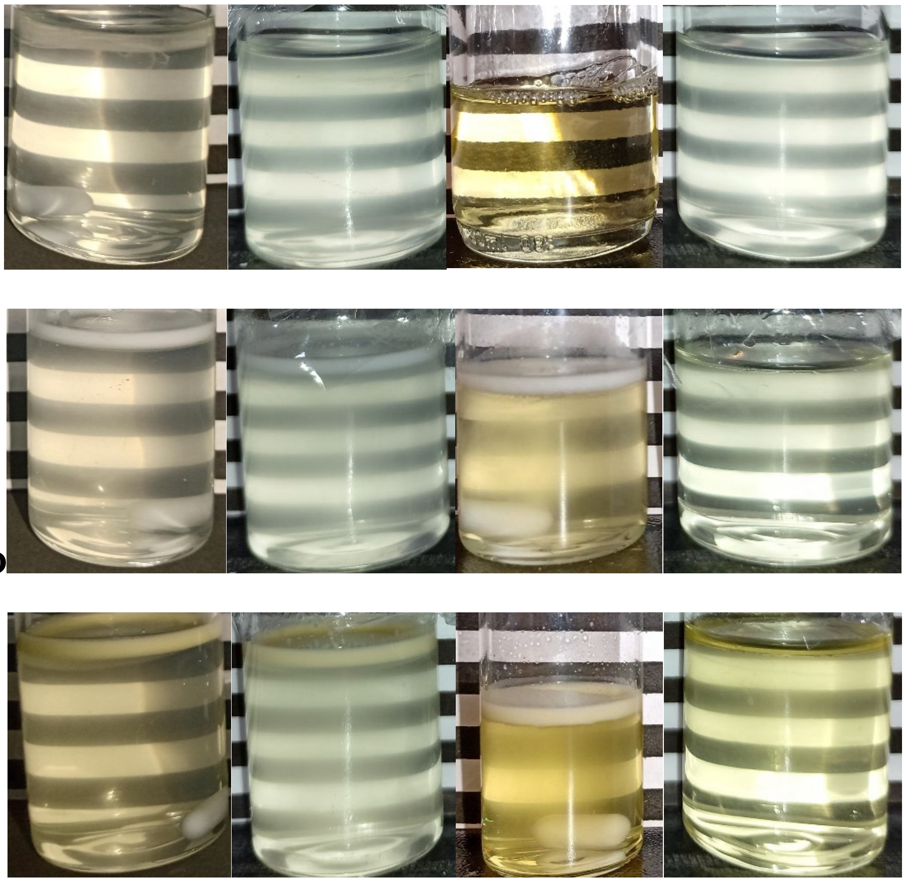

TTO

LavO

MO

Figure S5. Visual appearance of 5 wt% EOs emulsions prepared with 15 wt % PS60 solutions: first column: without additives; second column with 0.7M PG; third column 0.7 M KSorb; forth column: 0.7M CA+NaCitr.

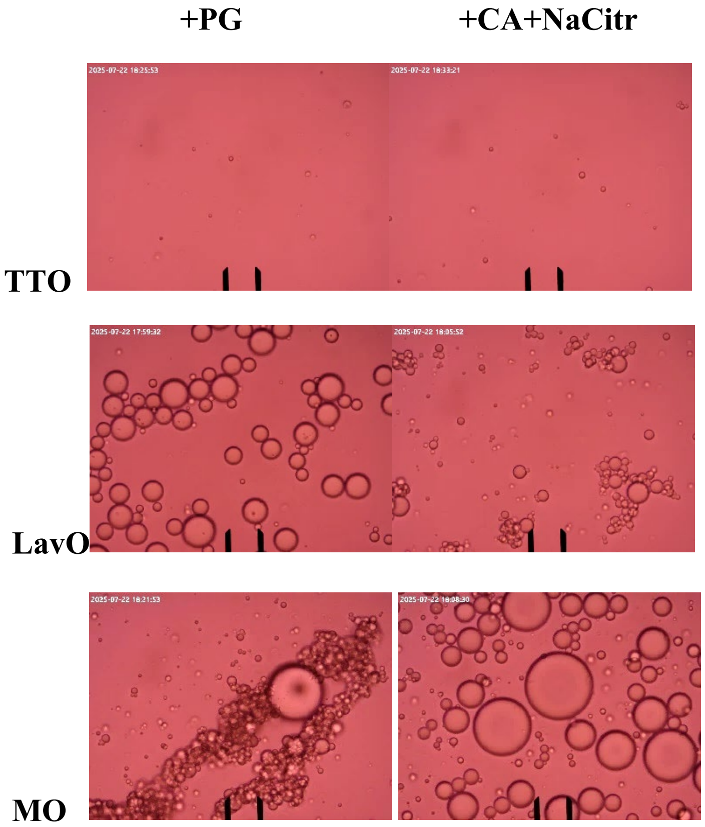

Figure S6. Microscope images of 0.7 M EOs emulsions prepared with 15 wt % PS60 solutions for TTO, LavO and MO emulsions prepared with a propylene glycol or citric acid + sodium citrate.
